# SKAP1 Expression in Cancer Cells Enhances Colon Tumor Growth and Impairs Cytotoxic Immunity by Promoting Neutrophil Extracellular Trap Formation via the NFATc1/CXCL8 Axis

**DOI:** 10.1002/advs.202403430

**Published:** 2024-09-13

**Authors:** Jian Gao, Jun Liu, Jilin Lu, Xiaofei Zhang, Wei Zhang, Qian Li, Jiayi Cai, Mengjun Li, Yu Gan, Yifan Tang, Shuangjie Wu

**Affiliations:** ^1^ State Key Laboratory of Systems Medicine for Cancer, Shanghai Cancer Institute Renji Hospital Shanghai Jiao Tong University School of Medicine Shanghai 200032 China; ^2^ Department of General Surgery Huashan Hospital (Hongqiao Campus) Fudan University Shanghai 201107 China; ^3^ Clinical Research Unit Renji Hospital Shanghai Jiao Tong University School of Medicine Shanghai 200127 China

**Keywords:** colon cancer, CXCL8, neutrophil extracellular trap, NFATc1, SKAP1

## Abstract

The mechanisms underlying the development and progression of colon cancer are not fully understood. Herein, Src kinase associated phosphoprotein 1 (SKAP1), an immune cell adaptor, is identified as a novel colon cancer‐related gene. SKAP1 expression is significantly increased in colon cancer cells. High SKAP1 levels are independently predictive of poor survival in patients with colon cancer. Notably, SKAP1 expression in colon cancer cells exerted a significant tumor‐promoting effect in vivo rather than in vitro. Screening of tumor‐infiltrating immune cells revealed the involvement of neutrophils in SKAP1‐induced colon tumor promotion. Enhanced formation of neutrophil extracellular traps (NETs) is found to be a key downstream event that contributed to the pro‐tumor role of SKAP1. In colon cancer cells, SKAP1 increased the expression of C‐X‐C motif chemokine ligand 8 (CXCL8) via nuclear factor of activated T cells c1 (NFATc1). The blockade of CXCL8 or NFATc1 largely attenuated neutrophil infiltration, NET formation, and tumor promotion induced by SKAP1. Furthermore, inhibiting SKAP1‐induced NET significantly enhanced the antitumor efficiency of adoptive natural killer cell therapy in colon tumor models. In conclusion, SKAP1 significantly promotes colon cancer growth via the cancer cell/neutrophil NFATc1/CXCL8/NET axis, suggesting that SKAP1 is a potential target for colon cancer therapy.

## Graphical abstract

Src kinase associated phosphoprotein 1 (SKAP1), an immune cell adaptor, is significantly upregulated in colon cancer cells, leading to increased C‐X‐C motif chemokine ligand 8 expression through  a nuclear factor of activated T cells c1‐dependent mechanism. This recruits neutrophils into the tumor microenvironment and promotes the formation of neutrophil extracellular traps, which potently enhance tumor growth and impair cytotoxic immunity, suggesting SKAP1 as a promising therapeutic target in colon cancer.

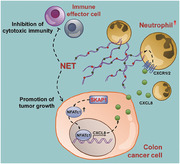

## 1 Introduction

Colon cancer is a frequently lethal disease of the digestive system. Over the last few years, colon cancer has remained the third most common malignancy in terms of incidence and mortality, affecting millions of people worldwide.^[^ ^1^ ^]^ Therefore, it is important to gain novel insights into the mechanisms that enable the development and malignant progression of colon cancer.

Over the past two decades, high‐throughput gene expression profiling has been widely used to identify potential cancer‐related genes whose expression levels are substantially altered in cancer tissues compared to that in corresponding noncancerous tissues. Analyses of the clinical relevance of the expression profiles of these genes have revealed valuable genetic markers that predict clinical outcomes independent of conventional risk factors such as disease stage.^[^ ^2^ ^]^ However, the biological functions of a considerable number of newly discovered predictor genes during cancer progression and their underlying mechanisms remain unclear. Using a similar strategy, the present study identified a novel colon cancer‐related gene, Src kinase‐associated phosphoprotein 1 (SKAP1), which was significantly upregulated in colon cancer and closely associated with the prognosis of patients with colon cancer. Importantly, our experiments further showed that SKAP1 displayed significant pro‐tumor activity in colon cancer and, interestingly, neutrophils were involved in the tumor‐promoting effect of SKAP1.

SKAP1, also known as SKAP‐55, was initially found to be predominantly expressed in T cells;^[^ ^3^ ^]^ therefore, most previous studies have focused on its physiological role in this type of lymphocytes. It has since been established that SKAP1 acts as an adaptor coupling the T‐cell receptor with “inside‐out” signaling for integrin‐mediated T‐cell adhesion, which is needed for the optimal antigen‐dependent activation of T cells.^[^ ^4^ ^]^ Its role in regulating T‐cell immunity has also been investigated under pathological conditions such as autoimmune diseases^[^ ^5^ ^]^ and cancers. In certain tumor models, SKAP1 deficiency was shown to suppress the expression of programmed cell death 1 (PD‐1) in cytotoxic T cells to enhance antitumor immunity.^[^ ^6^ ^]^ Intriguingly, apart from T‐cell lymphoma,^[^ ^7^ ^]^ increased SKAP1 expression was recently observed in non‐hematologic cancers, such as colorectal^[^ ^8^ ^]^ and gastric cancers.^[^ ^9^ ^]^ In addition, genomic structural analyses identified a SKAP1 fusion gene expressed in breast cancer cells,^[^ ^10^ ^]^ suggesting a potential role for SKAP1 in cancer cells. However, the functional effects and mechanisms involved have rarely been studied.

Neutrophils are the most abundant circulating leukocytes and play a critical role in the innate immune response during infection.^[^ ^11^ ^]^ Emerging evidence suggests that in several human cancers, including colon cancer, neutrophils are among the most prevalent immune cell types in the tumor microenvironment.^[^ ^12^ ^]^ Despite their undeniable antitumor functions, neutrophils have been largely suggested to elicit pro‐tumor activity via direct and indirect effects on malignant cells.^[^ ^13^ ^]^ In colorectal cancer, a high number of intratumoral neutrophils is associated with a malignant phenotype and has been identified as an independent predictor of adverse prognosis.^[^ ^14^ ^]^ Experimental studies have shown that neutrophils can stimulate cancer cell proliferation by releasing neutrophil extracellular traps (NETs), which are neutrophil‐derived DNA structures comprising decondensed chromatin filaments and granular proteins such as myeloperoxidase (MPO).^[^ ^15^ ^]^ Moreover, they suppressed T cell activity in the colon cancer microenvironment,^[^ ^16^ ^]^ suggesting that neutrophils are important contributors to the tumor‐promoting immune microenvironment. Although the immune context of the tumor microenvironment is believed to be shaped, in great part, by malignant cells,^[^ ^17^ ^]^ the molecular features of cancer cells that may facilitate neutrophil infiltration and cancer cell‐neutrophil crosstalk remain to be fully elucidated.

Herein, based on the clinical significance of SKAP1 revealed by our bioinformatics and tissue microarray analyses, we further experimentally determined the pro‐tumor function of SKAP1 in colon cancer and, for the first time, revealed SKAP1‐induced cellular interactions between cancer cells and neutrophils. SKAP1 expression in colon cancer cells significantly promoted neutrophil infiltration and NET formation, which largely contributed to the tumor‐promoting effect of SKAP1, via nuclear factor of activated T cells c1 (NFATc1)‐dependent C‐X‐C motif chemokine ligand 8 (CXCL8) expression. Furthermore, the inhibition of SKAP1‐induced NETs significantly enhanced the efficacy of adoptive immune cell therapy in colon cancer. Our findings suggest that the SKAP1‐NFATc1‐CXCL8‐NET axis is a novel mechanism underlying colon cancer progression, and that targeting SKAP1 is a potential strategy for colon cancer treatment.

## 2 Results

### 2.1 SKAP1 is Upregulated in Colon Cancer Cells, and High SKAP1 Expression Predicts Poor Prognosis in Patients with Colon Cancer

**Figure 1:**
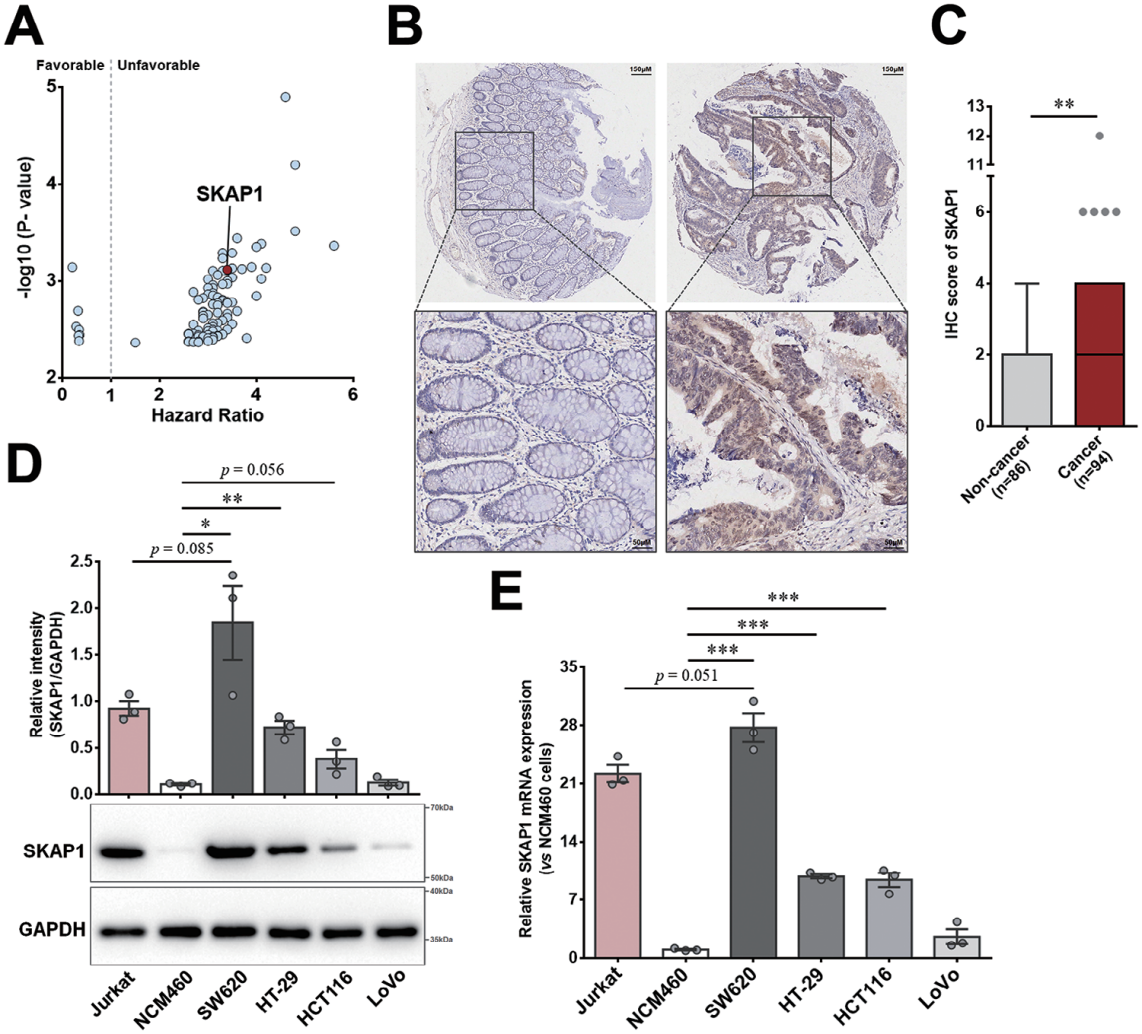
Increased SKAP1 expression in colon cancer cells. A) Plot diagram showing the hazard ratios (X‐axis) and *p* values (Y‐axis) of the top 100 genes with the lowest nominal *p* values in the association analyses between gene expression level and disease‐free survival in patients with colon cancer. Each dot represents one gene. These genes were identified using GEPIA2 online bioinformatics tools based on TCGA database. B‐C) Immunohistochemical analysis of a colon cancer tissue microarray section. B) Representative immunohistochemistry images of SKAP1 staining in colon cancer tissues and adjacent noncancerous colon tissues. C) Immunohistochemistry scores of SKAP1 staining for colon cancer (n = 94) and noncancerous tissues (n = 86). The *p* value was calculated using the Mann‐Whitney test. D) Western blot analysis of SKAP1 protein expression in colon cancer cell lines (SW620, HT‐29, HCT116, and LoVo), a normal colonic epithelial cell line (NCM460), and a leukemic T cell line (Jurkat). The histogram shows the densitometric quantification of the bands, and the representative blots are presented below. E) Quantitative PCR analyses of SKAP1 mRNA expression in the aforementioned cell lines. D‐E) Data are presented as the mean ± SEM. ***, *p* < 0.001; **, *p* < 0.01; *, *p* < 0.05.

Based on the data from The Cancer Genome Atlas (TCGA) database, we applied the “Most Differential Survival Genes” function of the Gene Expression Profiling Interactive Analysis 2 (GEPIA2)^[^ ^18^ ^]^ online bioinformatics tools to identify potential novel tumor progression‐associated genes in colon cancer. Among the top 100 genes that showed the lowest nominal *P* values in the association analyses of gene expression levels with disease‐free survival (**Figure** **1A**; Table S1, Supporting Information), SKAP1 was found to be a colon cancer‐associated candidate gene because: 1) SKAP1 expression was significantly higher in colon or colorectal cancers than in adjacent noncancerous tissues, as revealed by analyzing the data from both TCGA and Gene Expression Omnibus (GEO) databases (Figure S1A,B, Supporting Information) and 2) Cox regression analysis of the TCGA colon cancer cohort showed that high SKAP1 expression was an independent risk factor for poor overall survival (Figure S1C, Supporting Information).

To determine SKAP1 expression in colon cancer cells and further confirm the above results from RNA‐based bioinformatics analyses, immunohistochemical evaluation of SKAP1 was performed on a tissue microarray containing 94 colon cancer tissues and 86 adjacent noncancerous tissues. Although SKAP1 expression was barely detectable in normal colon epithelial cells, a significant proportion of colon cancer cells showed positive immunostaining for SKAP1 (Figure 1B). Quantitative analysis revealed that SKAP1 protein expression was significantly higher in cancer tissues than in noncancerous tissues (Figure 1C). Similar to our tissue microarray results, SKAP1 expression was significantly higher in three of the four tested human colon cancer cell lines than that in normal colonic epithelial NCM460 cells (Figure 1D,E). Interestingly, the colon cancer SW620 cells expressed higher levels of SKAP1 than Jurkat cells (Figure 1D,E), a human leukemic T‐cell line constitutively expressing SKAP1.^[^ ^19^ ^]^ This was supported by the analytical results of 985 cancer cell lines from the Human Protein Atlas portal, which showed that the average expression level of SKAP1 in colorectal cancer cells was comparable to that in lymphoma and leukemia cells (Figure S1D, Supporting Information). Together, these results reveal that SKAP1 expression is substantially increased in colon cancer cells, suggesting a potential oncogenic role of SKAP1 in colon cancer.

### 2.2 SKAP1 Potently Promotes Colon Tumor Growth In Vivo Rather Than In Vitro

**Figure 2:**
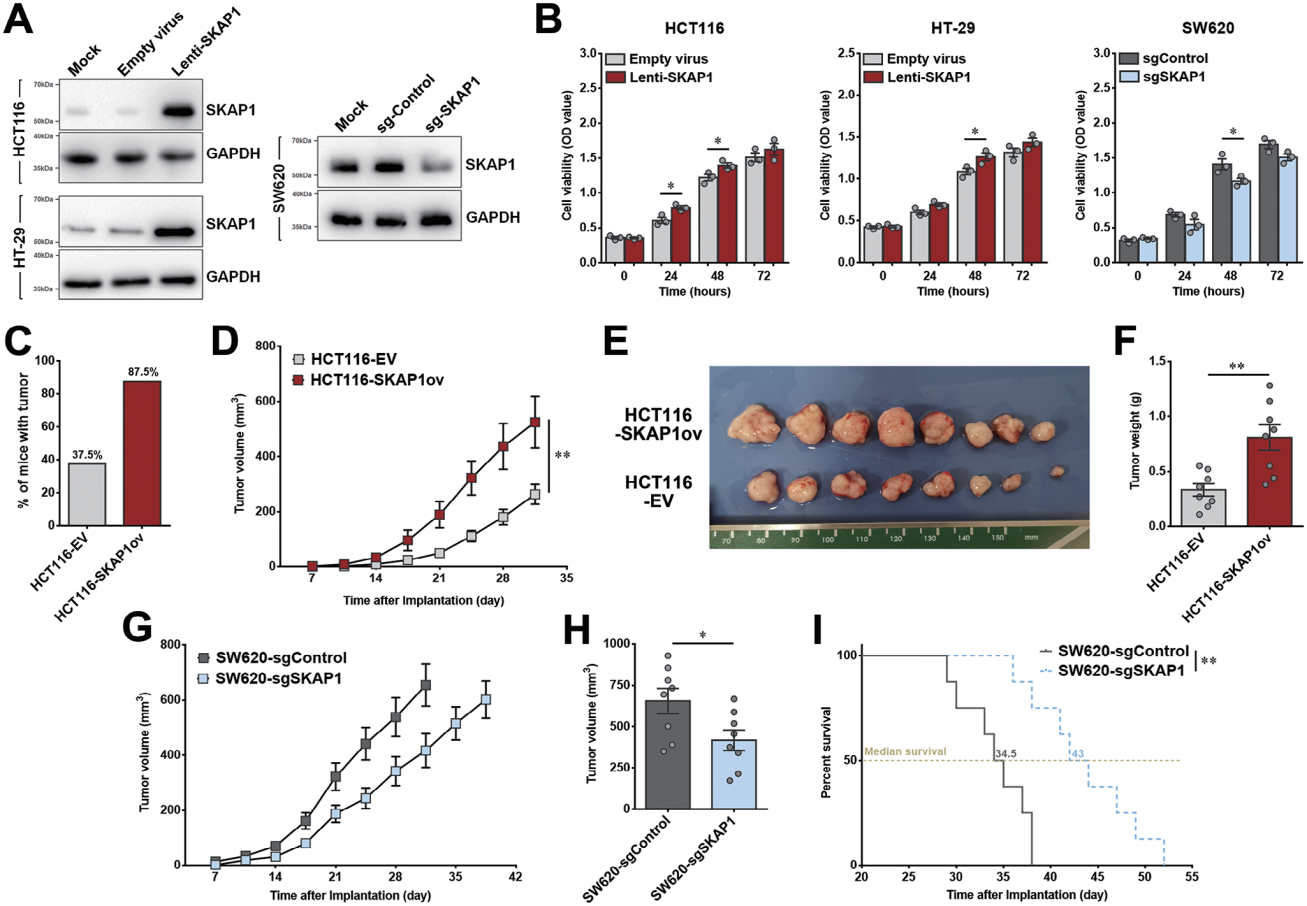
SKAP1 exerts a potent tumor‐promoting effect on colon cancer in vivo. HCT116 or HT‐29 cells were infected with a lentivirus carrying the SKAP1 gene (Lenti‐SKAP1) to generate SKAP1‐overexpressing cells. Empty virus was used as a control. SW620 cells were transfected with a CRISPR/Cas9 plasmid carrying the guide RNA targeting SKAP1 (sgSKAP1) to generate SKAP1‐knockdown cells or with a plasmid expressing a scrambled guide RNA (sgControl) as a control. A) Western blot analysis confirming the overexpression or knockdown of SKAP1 in the indicated colon cancer cells (representative blots of two independent experiments). B) The in vitro proliferation of SKAP1‐overexpressing or SKAP1‐knockdown cells was measured using CCK‐8 assay. C–F) Nude mice (n = 8 per group) were subcutaneously injected with SKAP1‐overexpressing HCT116 cells (HCT116‐SKAP1ov) or control cells (HCT116‐EV). C) Percentage of mice with visible tumors 7 days after inoculation. D) Tumor volumes in each group were monitored on the indicated days. E) Mice were sacrificed 31 days after tumor inoculation, and xenograft tumors were excised. F) Comparison of tumor weights between the HCT116‐SKAP1 and HCT116‐EV groups. G‐I) Nude mice (n = 8 per group) were subcutaneously injected with SKAP1‐knockdown SW620 cells (SW620‐sgSKAP1) or control cells (SW620‐sgControl). G) Tumor growth curves for each group. H) Comparison of tumor volume between SW620‐sgSKAP1 and SW620‐sgControl tumors 31 days after tumor inoculation. I) Survival curves of tumor‐bearing mice for each group. The mice were humanely sacrificed and declared dead when the tumor volume reached 800 mm^3^ or when body weight loss surpassed 20%. Data are presented as the mean ± SEM. **, *p* < 0.01; *, *p* < 0.05.

To determine the functional influence of SKAP1 on colon cancer, we overexpressed SKAP1 in HCT1116 and HT‐29 cells (**Figure** **2**A), which presented relatively low SKAP1 expression among the detected colon cancer cells (Figure 1E). The CCK‐8 assay showed that SKAP1 overexpression slightly promoted the proliferation of colon cancer cells in vitro (Figure 2B). Meanwhile, we silenced SKAP1 expression in SW620 cells (Figure 2A), which showed relatively high intrinsic SKAP1 expression (Figure 1E). SKAP1 knockdown only slightly slowed cell proliferation (Figure 2B). In addition, increased SKAP1 expression did not change the in vitro migration and invasion abilities of colon cancer cells (Figure S2, Supporting Information), suggesting that the direct influence of SKAP1 on colon cancer cells is limited.

Unexpectedly, we observed a potent in vivo tumor‐promoting effect of SKAP1 in nude mice. In a xenograft model of human HCT116 colon cancer cells, the occurrence of visible tumors was accelerated by SKAP1 overexpression, with 87.5% of mice (7/8) in the SKAP1‐overexpressing group (HCT116‐SKAP1ov) showing visible tumors 7 days after inoculation, whereas 62.5% of mice (5/8) in the control group (HCT116‐EV) still showed no tumors (Figure 2C). Moreover, SKAP1‐overexpressing tumors grew significantly faster than the control tumors (*P* < 0.01; Figure 2D). As a result, SKAP1 overexpression led to markedly larger tumors at the time of sacrifice (Figure 2E) and an almost 2.5‐fold increase in tumor weight (0.81 ± 0.16 g versus 0.33 ± 0.07 g, *P* < 0.01; Figure 2F). In contrast, SW620 colon tumors with SKAP1 knockdown (SW620‐sgSKAP1) showed slower in vivo growth kinetics (Figure 2G) and were significantly smaller than control tumors (SW620‐sgControl, *P* < 0.05; Figure 2H). Furthermore, mice bearing SKAP1‐knockdown tumors had substantially prolonged survival times compared to mice bearing control tumors (*P* < 0.01; Figure 2I).

### 2.3 Neutrophils are Involved in the Colon Tumor‐Promoting Effect of SKAP1

**Figure 3:**
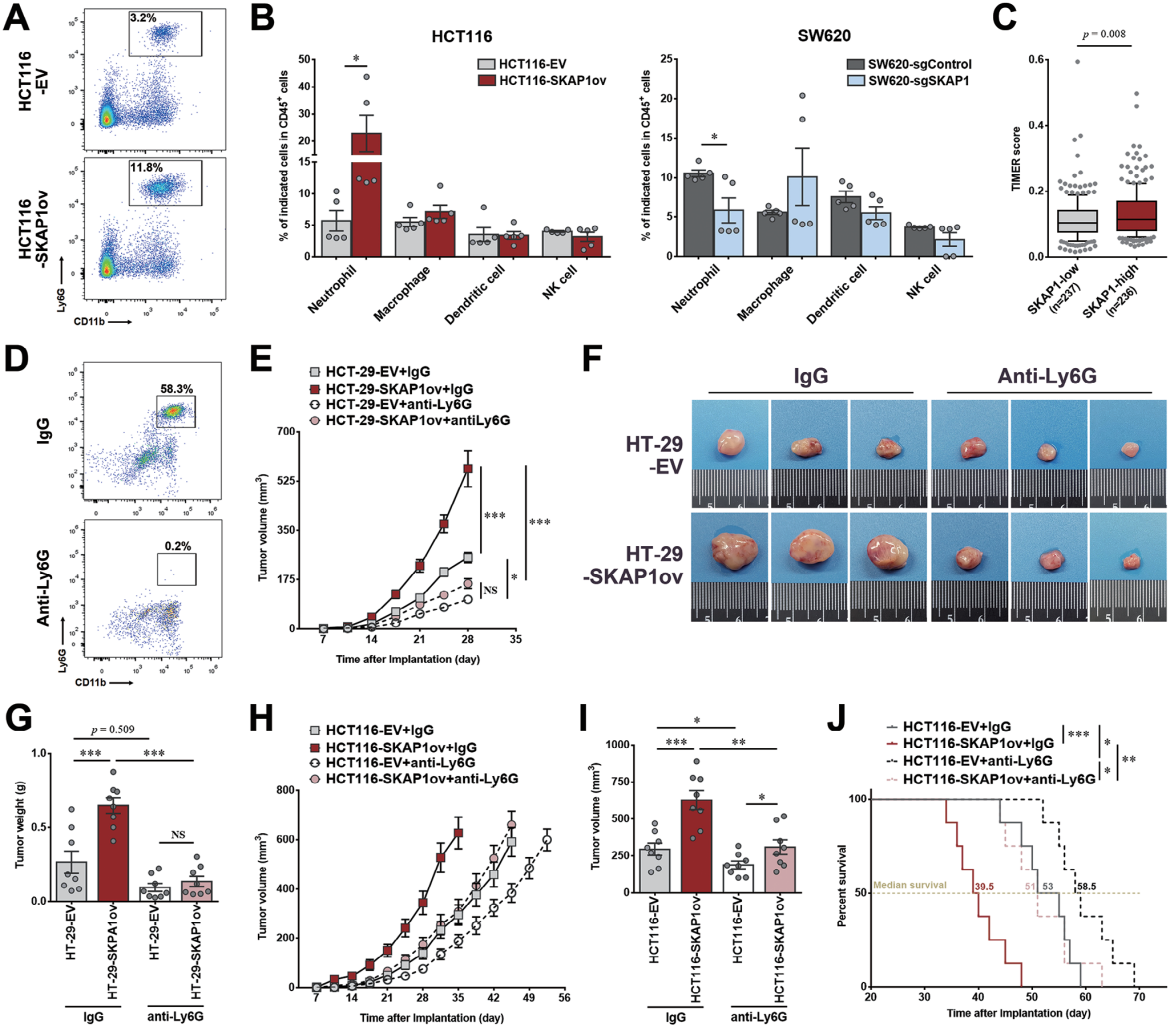
Neutrophils are important for the tumor‐promoting effects of SKAP1 in colon tumors. A‐B) Flow cytometric analysis of tumor‐infiltrating immune cells in subcutaneous colon tumors (n = 5 per group). Representative flow cytometry panels for the analysis of tumor‐infiltrating neutrophils (CD11b^+^ Ly6G^+^) in HCT116 tumors overexpressing SKAP1 (HCT116‐SKAP1ov) or control tumors (HCT116‐EV) are shown in A. B) Comparison of the percentages of indicated tumor‐infiltrating immune cells between HCT116‐SKAP1ov tumors or SKAP1‐knockdown SW620 tumors (SW620‐sgSKAP1) and their corresponding control tumors (HCT116‐EV or SW620‐sgControl). C) Neutrophil enrichment score estimated by the TIMER algorithm (TIMER score) for SKAP1‐high or SKAP1‐low (based on its median expression level) colon cancers from TCGA database. The *P* value was calculated using the Mann‒Whitney test. D‐I) For in vivo depletion of Ly6G^+^ cells, mice were injected with anti‐Ly6G or IgG control 2 days before the implantation of HT‐29 (HT‐29‐EV or HT‐29‐SKAP1ov) or HCT116 (HCT116‐EV or HCT116‐SKAP1ov) cells. D) The percentage of CD11b^+^ Ly6G^+^ cells in blood samples was analyzed using flow cytometry. Representative flow cytometry panels for 3 independent experiments are shown here. E) Comparison of the growth curves of HT‐29‐SKAP1ov and HT‐29‐EV tumors in anti‐Ly6G‐ or IgG‐treated mice (n = 8 per group). F) Twenty‐eight days after implantation of HT‐29 cells, the mice were sacrificed, and the tumors were dissected. Representative tumors from each group are shown. G) Comparison of HT‐29 tumor weights between groups. H) Comparison of the growth curves of HCT116‐SKAP1ov and HCT116‐EV tumors in anti‐Ly6G‐ and IgG‐treated mice (n = 8 per group). I) Comparison of HCT116 tumor volumes between groups 35 days after tumor inoculation. J) Survival curves of HCT116 tumor‐bearing mice for each group. The mice were humanely sacrificed and declared dead when the tumor volume reached 800 mm^3^ or when body weight loss surpassed 20%. B, E and G‐I) Data are presented as the mean ± SEM. ***, *p* < 0.001; **, *p* < 0.01; *, *p* < 0.05; NS, *p* > 0.05.

The observation that SKAP1 elicits a more powerful tumor‐promoting effect in vivo than in vitro suggests the possible involvement of noncancerous cells in SKAP1‐induced colon tumor promotion. To test this hypothesis, we analyzed various tumor‐infiltrating immune cells using flow cytometry and found that there were significantly more CD11b^+^ Ly6G^+^ neutrophils, rather than other detected immune cells, in SKAP1‐overexpressing HCT116 tumors (**Figure** **3**A,B; Figure S3A,B, Supporting Information). In contrast, the proportion of neutrophils was markedly decreased by SKAP1 knockdown in SW620 tumors (Figure 3B), suggesting that SKAP1 expression in cancer cells promotes neutrophil infiltration into colon tumors. This was supported by TIMER algorithm‐based analysis using TCGA database, which showed that colon cancer samples with high SKAP1 expression exhibited significantly higher neutrophil enrichment scores than those with low SKAP1 expression (Figure 3C). Samples with high SKAP1expression also exhibited higher enrichment scores for CD8^+^ T cells (Figure S3C, Supporting Information), possibly because T cells express relatively high levels of SKAP1.

A recent study demonstrated the coexistence of classical neutrophils and polymorphonuclear myeloid‐derived suppressor cells (PMN‐MDSCs), which are considered as pathologically activated neutrophils,^[^ ^20^ ^]^ in tumors and identified CD14 as a marker to distinguish PMN‐MDSCs (Ly6G^+^ CD14^+^) from classical neutrophils (Ly6G^+^ CD14^−^).^[^ ^21^ ^]^ Notably, the proportion of not only CD14^−^ neutrophils but also CD14^+^ PMN‐MDSCs in total tumor‐infiltrating CD45^+^ cells was significantly increased by SKAP1 overexpression (Figure S3D,E, Supporting Information), suggesting the accumulation of PMN‐MDSCs in SKAP1‐overexpressing colon tumors. Nonetheless, CD14^−^ neutrophils were the predominant subpopulation in tumor‐infiltrating CD11b^+^ Ly6G^+^ cells (> 70%) regardless of whether SKAP1 was overexpressed and there was no difference in the proportion of CD14^+^ PMN‐MDSCs in CD11b^+^ Ly6G^+^ cells between the SKAP1‐overexpressing and control groups (Figure S3D,F, Supporting Information). Additionally, CD11b^+^ Ly6G^+^ cells from SKAP1‐overexpressing tumors expressed similar levels of arginase 1, an immunosuppressive enzyme that is markedly upregulated in PMN‐MDSCs,^[^ ^22^ ^]^ as those from control tumors (Figure S3G, Supporting Information).

We then depleted Ly6G^+^ cells in tumor‐bearing mice by injecting Ly6G‐specific antibodies, and successful depletion of Ly6G^+^ cells was confirmed using flow cytometry (Figure 3D). Ly6G^+^ cell depletion significantly inhibited colon tumor growth regardless of whether SKAP1 was overexpressed in cancer cells (Figure 3E–I), underscoring the tumor‐promoting effects of neutrophils in our experiments. Importantly, in mice in which Ly6G^+^ cells were depleted, the growth‐promoting effect of SKAP1 on colon tumor xenografts was substantially attenuated (Figure 3E–I). SKAP1 overexpression led to a more than 2.4‐fold increase in HT‐29 tumor weight in IgG‐treated mice (0.65 ± 0.05 g versus 0.26 ± 0.08 g, *P* < 0.001) 28 days after tumor implantation, but failed to significantly increase tumor weights in anti‐Ly6G antibody‐treated mice (*P* > 0.05; Figure 3G). Similar results were observed in the HCT116 colon cancer model (Figure 3H,I). Furthermore, Ly6G^+^ cell depletion prolonged the survival of HCT116 tumor‐bearing mice and substantially weakened the adverse effects of SKAP1 overexpression on the survival of tumor‐bearing mice (Figure 3J), suggesting that SKAP1‐induced colon tumor promotion depends largely on the pro‐tumor activity of neutrophils.

### 2.4 NETs Contribute to the Enhanced Colon Tumor Growth Caused by SKAP1

**Figure 4:**
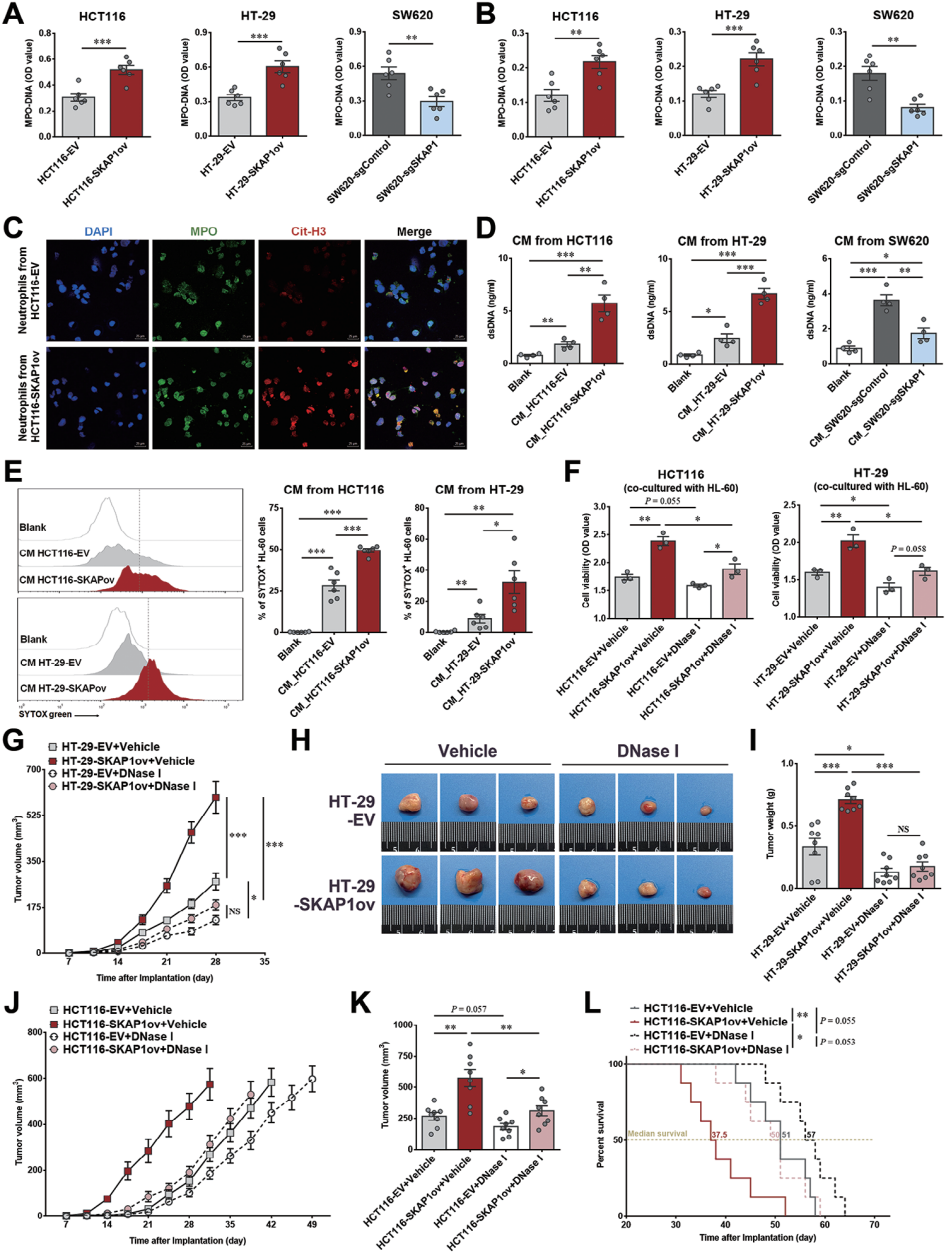
NETs contribute to enhanced colon tumor growth caused by SKAP1. A) MPO‐DNA levels in tumor tissue homogenates derived from SKAP1‐overexpressing (HCT116‐SKAP1ov and HT‐29‐SKAP1ov) or SKAP1‐knockdown (SW620‐sgSKAP1) tumors and their corresponding control tumors (HCT116‐EV, HT‐29‐EV, or SW620‐sgControl). B) Plasma MPO‐DNA levels in tumor‐bearing mice. A,B) n = 6 per group. C) Immunofluorescence staining for MPO (green) and the presence of NETs (Cit‐H3, red) in neutrophils isolated from HCT116‐SKAP1ov and HCT116‐EV tumors. Representative images are shown, and similar images were obtained from the tumor‐infiltrating neutrophils of 4 mice in each group. D,E) Neutrophil‐differentiated HL‐60 cells were cultured with CM from SKAP1‐overexpressing (HCT116‐SKAP1ov and HT‐29‐SKAP1ov) or SKAP1‐knockdown cells (SW620‐sgSKAP1) and their corresponding control cells (HCT116‐EV, HT‐29‐EV, or SW620‐sgControl). D) Detection of cell‐free double‐stranded DNA levels in culture supernatants. E) Flow cytometry analyses of NET‐forming (SYTOX green^+^) HL‐60 cells after culturing with the indicated CM. Representative flow cytometry histograms are shown in left panel. F) CCK‐8 assay for cell proliferation of SKAP1‐overexpressing (HCT116‐SKAP1ov or HT‐29‐SKAP1ov) or control cells (HCT116‐EV or HT‐29‐EV) co‐cultured with neutrophil‐differentiated HL‐60 cells in the presence or absence of DNase I. G–L) Mice bearing SKAP1‐overexpressing colon tumors (HT‐29‐SKAP1ov or HCT116‐SKAP1ov) or control tumors (HT‐29‐EV or HCT116‐EV) were treated with DNase I or the same volume of saline (vehicle groups). G) Comparison of the growth curves of HT‐29‐SKAP1ov and HT‐29‐EV tumors in DNase I‐ or saline‐treated mice (n = 8 per group). H) Representative HT‐29 tumors from each group. I) Comparison of HT‐29 tumor weights between groups. J) Comparison of the growth curves of HCT116‐SKAP1ov and HCT116‐EV tumors in DNase I‐ or saline‐treated mice (n = 8 per group). K) Comparison of HCT116 tumor volumes between groups 31 days after tumor inoculation. L) Survival curves of HCT116 tumor‐bearing mice for each group. The mice were humanely sacrificed and declared dead when the tumor volume reached 800 mm^3^ or when body weight loss surpassed 20%. Data are presented as the mean ± SEM. ***, *p* < 0.001; **, *p* < 0.01; *, *p* < 0.05; NS, *p* > 0.05.

We then investigated which specific function of neutrophils is responsible for the tumor‐promoting effect of SKAP1. Because NETs mediate the pro‐tumor activity of neutrophils,^[^ ^23^ ^]^ we investigated a potential functional link between cancer cell expression of SKAP1 and NET formation. The MPO‐DNA complex is considered as a marker of NETs.^[^ ^24^ ^]^ There were significantly higher MPO‐DNA levels in SKAP1‐overexpressing HCT116 and HT‐29 colon tumors than in control tumors (**Figure** **4**A). Additionally, plasma MPO‐DNA levels increased in mice bearing SKAP1‐overexpressing tumors (Figure 4B). In contrast, the tumor and plasma levels of MPO‐NDA were significantly decreased by SKAP1 knockdown in a mouse model of SW620 colon cancer (Figure 4A,B). The enhanced formation of NETs in SKAP1‐overexpressing tumors was further confirmed by immunofluorescence staining for citrullinated histone H3 (Cit‐H3), another specific marker of NETs, in tumor‐infiltrating neutrophils (Figure 4C). Through in vitro NETosis assays using neutrophil‐differentiated HL‐60 cells, we found that conditioned medium (CM) from SKAP1‐overexpressing colon cells was more potent, whereas CM from SKAP1‐knockdown cells was less potent in inducing NETosis, as determined by the detection of extracellular dsDNA (Figure 4D). The proportion of NET‐forming HL‐60 cells, as determined by flow cytometric analysis of SYTOX green‐positive cells,^[^ ^25^ ^]^ was also significantly increased by CM from SKAP1‐overexpressing colon cancer cells (Figure 4E). Together, these results suggest that increased SKAP1 expression in colon cancer cells promotes NET formation possibly via soluble molecules from cancer cells.

To determine whether NETs contribute to SKAP1‐induced colon tumor promotion, DNase I was used to degrade NET‐associated DNA. In an in vitro cancer cell/neutrophil co‐culture model, SKAP1‐overexpressing colon cancer cells exhibited a markedly increased proliferative capacity when co‐cultured with neutrophil‐differentiated HL‐60 cells, whereas DNase I treatment largely abolished this SKAP1‐induced proliferation enhancement (Figure 4F). We also observed similar results in vivo. Systemic DNase I treatment inhibited the growth of HT‐29 and HCT116 tumors in nude mice and largely attenuated the growth enhancement of colon tumors induced by SKAP1 overexpression (Figure 4G‐K). Additionally, NET degradation by DNase I substantially reduced the SKAP1 overexpression‐induced survival disadvantage in HCT116 tumor‐bearing mice (Figure 4L). These data suggested that colon cancer cell expression of SKAP1 promotes tumor growth largely by inducing NET formation.

### 2.5 SKAP1 Expression in Colon Cancer Cells Promotes NET Formation by Increasing CXCL8 Expression

**Figure 5:**
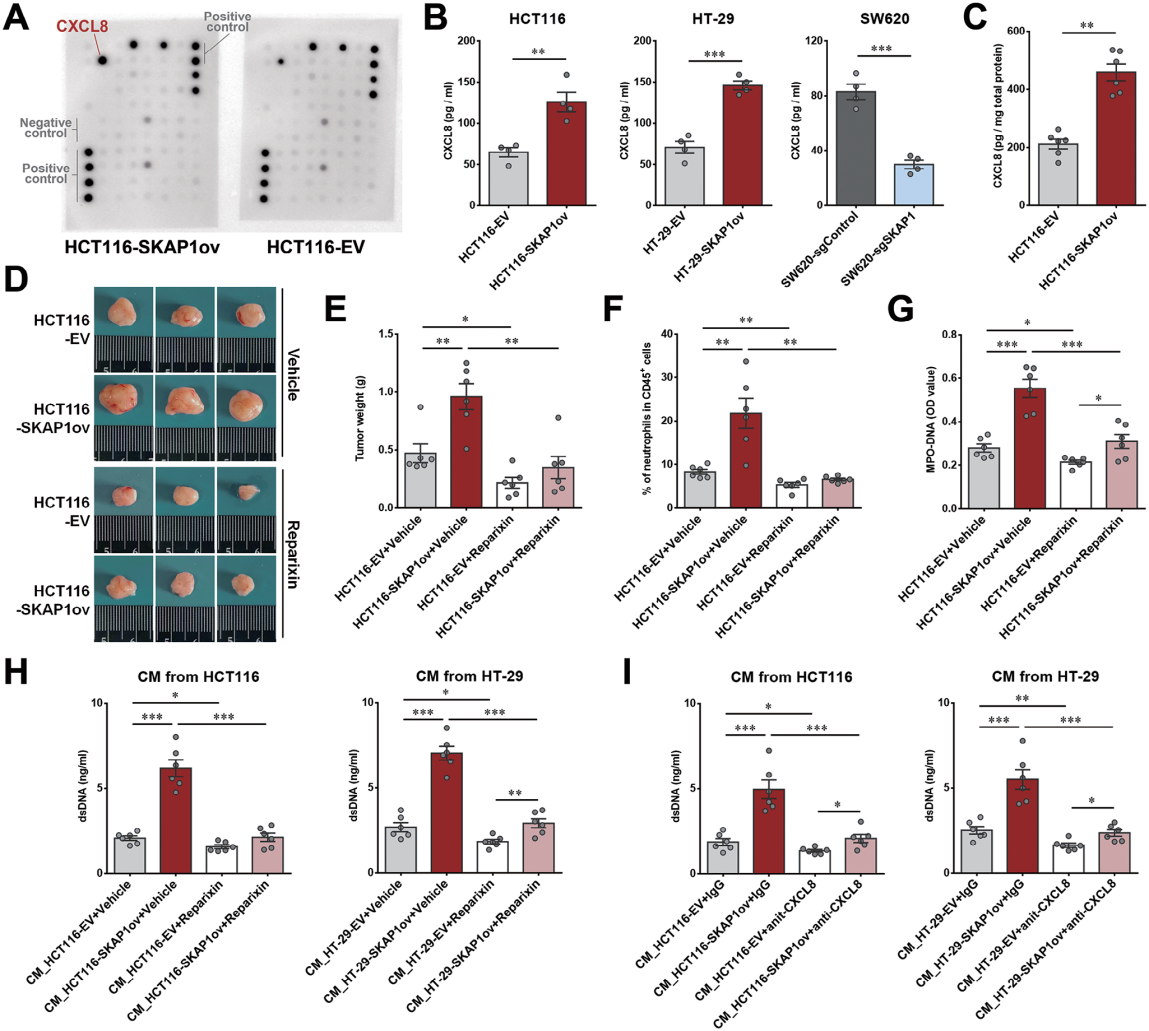
SKAP1 expression in colon cancer cells promotes neutrophil infiltration and NET formation via CXCL8. A) Cytokine array analysis of 80 secreted proteins in the CM of SKAP1‐overexpressing (HCT116‐SKAP1ov) and control HCT116 cells (HCT116‐EV). B) ELISA analysis of CXCL8 protein levels in the culture supernatants of SKAP1‐overexpressing (HCT116‐SKAP1ov and HT‐29‐SKAP1ov) or SKAP1‐knockdown cells (SW620‐sgSKAP1) and their corresponding control cells (HCT116‐EV, HT‐29‐EV, or SW620‐sgControl). C) ELISA analysis of CXCL8 levels in subcutaneous tumors formed by HCT116‐SKAP1ov and HCT116‐EV cells. D–G) Mice bearing HCT116‐EV or HCT116‐SKAP1ov tumors were treated with reparixin or the same volume of saline (vehicle groups). Thirty‐five days after tumor inoculation, the mice were sacrificed, and the tumors were dissected (n = 6 per group). Representative tumors for each group are shown in D. E) Comparison of tumor weights between groups. F) Flow cytometry analysis of the percentage of tumor‐infiltrating neutrophils in each group. G) The intratumoral levels of MPO‐DNA were detected using tumor homogenates. H,I) In vitro NETosis assays using CM from SKAP1‐overexpressing (HCT116‐SKAP1ov or HT‐29‐SKAP1ov) or control cells (HCT116‐EV or HT‐29‐EV) were performed to assess the influence of reparixin (H) and a CXCL8‐neutralizing antibody (I) on SKAP1‐induced NET formation. NET levels were determined by the detection of cell‐free dsDNA or MPO‐DNA complexes (see Figure S4C,D, Supporting Information) in culture supernatants. Data are presented as the mean ± SEM. ***, *p* < 0.001; **, *p* < 0.01; *, *p* < 0.05.

Soluble molecules may link SKAP1 expression in colon cancer cells and NET formation, therefore, we used a cytokine array to compare the levels of 80 secreted proteins in the CM of SKAP1‐overexpressing and control HCT116 cells. Among the 80 proteins detected, CXCL8 was the most upregulated, showing an approximately two‐fold increase (**Figure** **5**A; Figure S4A, Supporting Information). ELISA confirmed that SKAP1‐overexpressing HCT116 and HT‐29 cells secreted more CXCL8, whereas SKAP1‐knockdown SW620 cells secreted less CXCL8 than their corresponding control cells (Figure 5B). Compared to control tumors, CXCL8 displayed more than a two‐fold increase in SKAP1‐overexpressing HCT‐116 tumors (Figure 5C). In parallel, we analyzed the mRNA expression of several neutrophil recruitment‐ or NET‐related cytokines, including CXCL1, CXCL2, CXCL5, CXCL6, CXCL8, transforming growth factor‐β 1 (TGF‐β1), and tissue inhibitor of metalloproteinases‐1 (TIMP‐1),^[^ ^26^, ^27^, ^28^ ^]^ and found that only *CXCL8* mRNA levels were significantly increased by SKAP1 overexpression (Figure S4B, Supporting Information). These results suggested that SKAP1 promotes CXCL8 expression in colon cancer cells.

CXCL8, which is also known as interleukin (IL)−8, plays an important role in neutrophil recruitment and NET formation.^[^ ^29^ ^]^ Despite the absence of the CXCL8 homolog in mice, human CXCL8 can act on mouse cells via murine C‐X‐C motif chemokine receptor (CXCR) 1 and CXCR2.^[^ ^30^ ^]^ We then used the CXCR1/R2 inhibitor reparixin^[^ ^30^ ^]^ to block CXCL8 signaling. Systemic reparixin treatment largely attenuated the tumor‐promoting effect of SKAP1 (Figure 5D,E) and completely abolished SKAP1‐associated infiltration of neutrophils in HCT116 tumors (Figure 5F), suggesting that SKAP1 promotes neutrophil infiltration into colon tumors via CXCL8. In addition, SKAP1 overexpression‐enhanced MPO‐DNA level in HCT116 tumors was largely alleviated by reparixin treatment (Figure 5G). To further determine the role of CXCL8 in SKAP1‐induced NET formation, we performed in vitro NETosis assay. Both reparixin and CXCL8‐neutralizing antibodies efficiently inhibited the NET‐enhancing effect of CM from SKAP1‐overexpressing colon cancer cells (Figure 5H,I; Figure S4C,D, Supporting Information). Taken together, these results suggest that CLXCL8 is a key mediator of SKAP1‐induced neutrophil infiltration and NET formation in colon cancer.

### 2.6 SKAP1 is Dependent on NFATc1 to Promote CXCL8 Expression in Colon Cancer Cells

**Figure 6:**
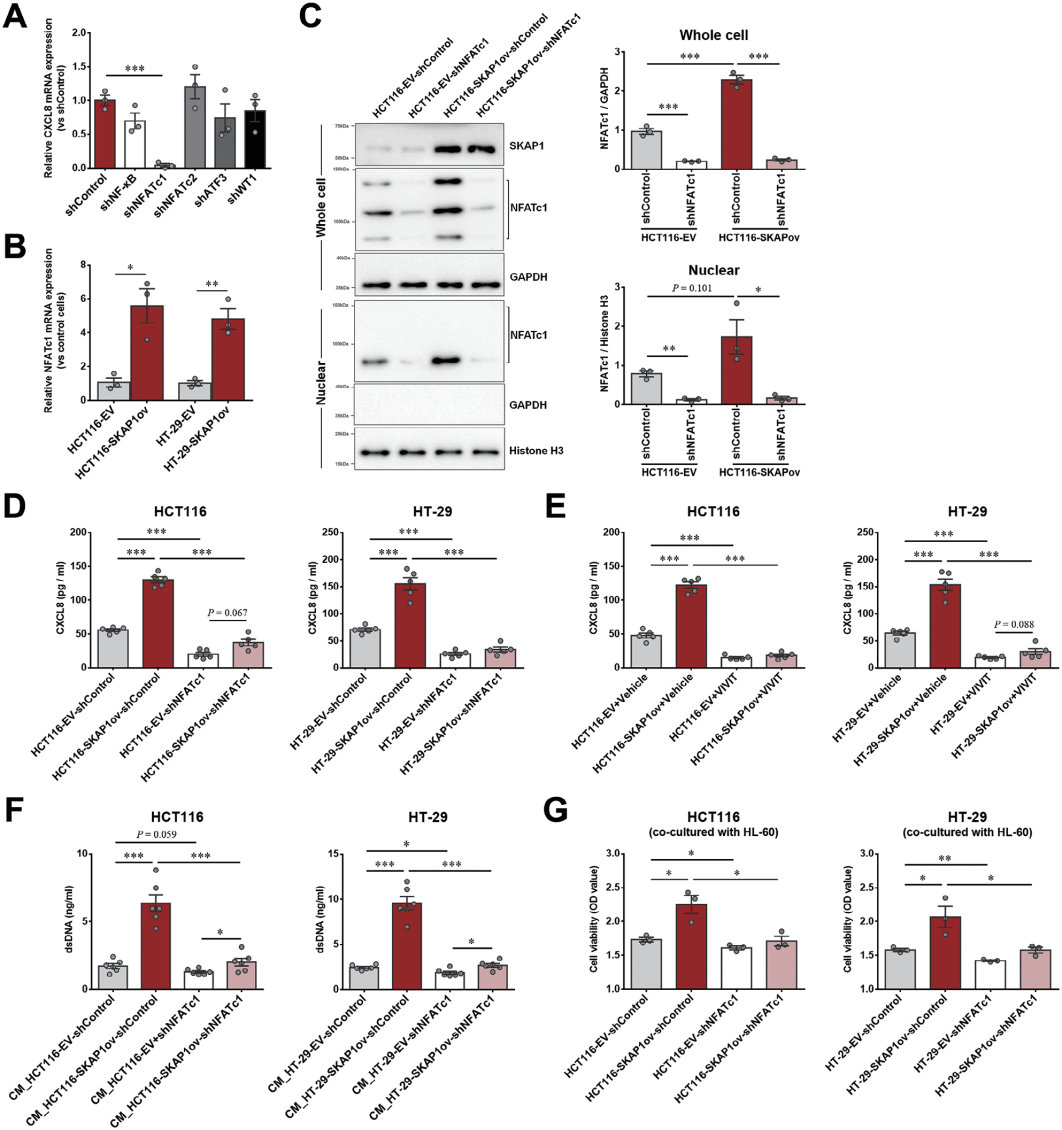
SKAP1 promotes CXCL8 expression via NFATc1 in colon cancer cells. A) Quantitative PCR analysis of *CXCL8* mRNA expression in HCT116‐SKAP1ov cells transfected with shRNA targeting the indicated transcription factors (shNF‐κB, shNFATc1, shNFATc2, shATF3, and shWT1) or a scrambled shRNA (shControl). B) Quantitative PCR analysis of *NFATc1* mRNA expression in SKAP1‐overexpressing (HCT116‐SKAP1ov or HT‐29‐SKAP1ov) and control colon cancer cells (HCT116‐EV or HT‐29‐EV). C) Western blot analyses of NFATc1 protein levels in whole‐cell lysates or nuclear fractions of HCT116‐SKAP1ov and HCT116‐EV cells transfected with shNFATc1 or shControl. Note that activated NFATc1 moved faster in SDS‐PAGE gels than the inactive form because of the dephosphorylation of multiple phosphoserines on NFATc1, which is required for its nuclear translocation. Quantitative data are illustrated in the right panel. D) ELISA analysis of CXCL8 levels in the culture supernatants of SKAP1‐overexpressing (HCT116‐SKAP1ov or HT‐29‐SKAP1ov) or control colon cancer cells (HCT116‐EV or HT‐29‐EV) transfected with shNFATc1 or shControl. E) ELISA analysis of CXCL8 levels in the culture supernatants of SKAP1‐overexpressing (HCT116‐SKAP1ov or HT‐29‐SKAP1ov) or control colon cancer cells (HCT116‐EV or HT‐29‐EV) treated with or without the NFAT inhibitory peptide VIVIT. F) In vitro NETosis assay using CM from SKAP1‐overexpressing (HCT116‐SKAP1ov or HT‐29‐SKAP1ov) or control cells (HCT116‐EV or HT‐29‐EV) transfected with shNFATc1 or shControl. NET levels were determined by detecting cell‐free dsDNA or MPO‐DNA complexes (see Figure S5B, Supporting Information) in culture supernatants. G) CCK‐8 assay to evaluate the cell proliferation of SKAP1‐overexpressing (HCT116‐SKAP1ov or HT‐29‐SKAP1ov) or control cells (HCT116‐EV or HT‐29‐EV) transfected with shNFATc1 or shControl in the presence of neutrophil‐differentiated HL‐60 cells. Data are presented as the mean ± SEM. ***, *p* < 0.001; **, *p* < 0.01; *, *p* < 0.05.

Transcription factors nuclear factor kappa B (NF‐κB),^[^ ^31^ ^]^ NFATc1,^[^ ^32^ ^]^ NFATc2,^[^ ^33^ ^]^ activating transcription factor 3 (ATF3),^[^ ^34^ ^]^ and Wilms’ tumor 1 (WT1)^[^ ^35^ ^]^ have been reported to regulate CXCL8 expression. We then investigated which transcription factor cooperates with SKAP1 to enhance CXCL8 expression in colon cancer cells. We separately knocked down these transcription factors in HCT116‐SKAP1ov cells (Figure S5A, Supporting Information) and found that only NFATc1 knockdown significantly decreased *CXCL8* mRNA expression (**Figure** **6**A). Moreover, *NFATc1* mRNA levels were significantly elevated by SKAP1 overexpression in colon cancer cells (Figure 6B). Western blot assays also showed that SKAP1 overexpression elevated the total NFATc1 protein levels in whole‐cell lysates and activated NFATc1 levels in nuclear lysates; these elevations were abolished by NFATc1 knockdown (Figure 6C). NFATc1 knockdown significantly decreased CXCL8 levels in the supernatants of colon cancer cells, whereas SKAP1 overexpression failed to significantly increase CXCL8 levels after NFATc1 knockdown (Figure 6D). Consistently, treatment with the NFAT inhibitory peptide VIVIT almost completely abolished SKAP1‐induced CXCL8 expression (Figure 6E), suggesting that SKAP1 dependents on NFATc1 to increase CXCL8 expression in colon cancer cells. Additionally, NFATc1 knockdown in colon cancer cells largely abolished the NET‐enhancing effect of CM from SKAP1‐overexpressing cells in the in vitro NETosis assay (Figure 6F; Figure S5B, Supporting Information) and attenuated SKAP1‐induced proliferation enhancement of colon cancer cells in the cancer cell/HL‐60 co‐culture models (Figure 6G), suggesting the involvement of NFATc1‐dependent regulation of CXCL8 in the tumor‐promoting effect of SKAP1.

### 2.7 Inhibition of SKAP1‐Induced NET Formation Enhanced the Antitumoral Efficiency of Natural Killer (NK) Cell Immunotherapy

**Figure 7:**
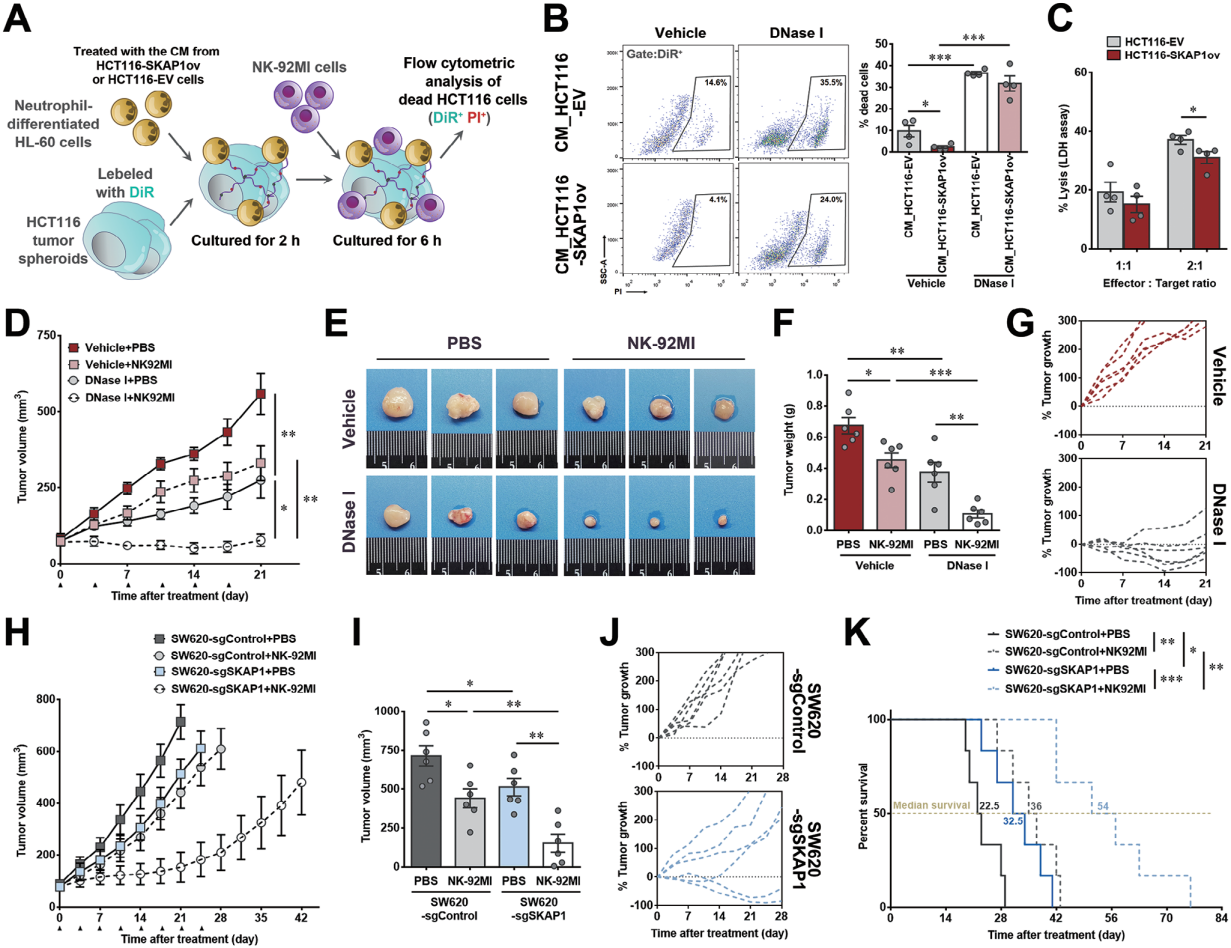
Inhibition of SKAP1‐induced NET formation improves therapeutic effects of NK cell therapy in colon cancer. A) Graphic scheme of the experiments as in (B). HCT116 tumor spheroids were labeled with DiR and cultured with neutrophil‐differentiated HL‐60 cells pre‐treated with the CM from SKAP‐overexpressing HCT116 cells (HCT116‐SKAP1ov) or control cells (HCT116‐EV). The spheroids and HL‐60 cells were subsequently co‐cultured with NK‐92MI cells. B) Flow cytometry analysis of dead HCT116 cells (DiR^+^ PI^+^). Representative flow cytometry panels are shown on the left side. C) LDH release assay to analyze the cytotoxicity of NK‐92MI cells against HCT116‐SKAP1ov or HCT116‐EV in the absence of HL‐60 cells. D‐G) NPG mice were subcutaneously injected with HCT116‐SKAP1ov cells and treated with NK‐92MI cells together with or without DNase I (n = 6 per group). The mean pre‐treatment tumor volume was ≈75 mm^3^ in each group, with no significant differences between the groups. D) Summary of follow‐up tumor growth measurements for each group. NK‐92MI cells or PBS were injected at the time points indicated by arrows. E) Twenty‐one days after treatment, the mice were sacrificed, and the tumors were dissected. Representative tumors from each group are shown. F) Comparison of tumor weights between groups. G) Individual tumor growth curves for Vehicle+NK‐92MI and DNase I+NK‐92MI groups. The results are shown that tumor volume changes compared to their initial size. H‐K) NPG mice were subcutaneously injected with SKAP1‐knockdown SW620 cells (SW620‐sgSKAP1) or control cells (SW620‐sgControl) and peritumorally administered with NK‐92MI cells or PBS after tumor formation (n = 6 per group). The mean pretreatment tumor volume was ≈75 mm^3^ in each group, with no significant differences between the groups. H) Summary of the follow‐up tumor growth measurements for each group. NK‐92MI cells or PBS were injected at the time points indicated by arrows. I) Comparison of tumor volumes between groups 21 days after the first injection of NK‐92MI cells or PBS. J) Individual tumor growth curves after NK‐92MI treatment for SW620‐sgControl and SW620‐sgSKAP1 tumors. The results are shown that tumor volume changes compared to their initial size. K) Survival curves of tumor‐bearing mice for each group. The mice were humanely sacrificed and declared dead when the tumor volume reached 800 mm^3^ or when body weight loss surpassed 20%. Data are presented as the mean ± SEM. ***, *p* < 0.001; **, *p* < 0.01; *, *p* < 0.05.

NETs can impair cytotoxic immunity mediated by immune effector cells, such as NK cells, against tumor cells.^[^ ^28^ ^]^ Given that SKAP1 expression in cancer cells significantly induced NET formation in our colon tumor models, we postulated that SKAP1 expression might impair immune‐mediated cytotoxicity in colon cancer. This hypothesis was supported by the co‐culture experiments of colon cancer cells with neutrophils and NK cells. As schematized in **Figure** **7**A, tumor spheroids derived from HCT116 cells were co‐cultured with neutrophil‐differentiated HL‐60 cells, which were pretreated with CM from SKAP1‐overexpressing HCT116 or control cells. Spheroids and neutrophils cultured in the presence or absence of DNase I were then co‐cultured with NK‐92MI cells. The proportion of dead cells in HCT116 cells was dramatically lower when co‐cultured with HL‐60 cells pretreated with CM from SKAP1‐overexpressing cells compared to HL‐60 cells pretreated with control cells’ CM (2.3 ± 0.6% versus 9.7 ± 2.7%; Figure 7B). In parallel, we also assessed the cytotoxicity of NK cells without co‐culturing with neutrophils and found that SKAP1 overexpression only slightly reduced NK‐92MI‐mediated cell lysis of HCT116 cells in the absence of neutrophils (Figure 7C), suggesting that SKAP1 confers resistance to cytotoxic NK cells in colon cancer cells mainly through neutrophils. NET degradation by DNase I markedly increased the sensitivity of colon cancer cells to NK‐cell cytotoxicity and abolished SKAP1‐induced resistance to NK cell cytotoxicity (Figure 7B), indicating an important contribution of NET to SKAP1‐induced immune resistance.

We further investigated the influence of NET inhibition on the therapeutic efficacy of adoptive NK cell treatment in SKAP1‐high colon tumors. NPG mice bearing SKAP1‐overexpressing HCT116 tumors were injected with NK‐92MI cells together with or without DNase I. NK cell treatment slowed the growth of SKAP1‐overexpressing HCT116 tumors, as revealed by tumor volume and weight measurements (Figure 7D–F). However, tumor regression, defined as a greater than 50% decrease in tumor mass when compared to its initial size, was not observed in any mouse treated with NK‐cell mono‐treatment (Figure 7G). DNase I treatment substantially increased the sensitivity of colon tumor xenografts to NK cell therapy (Figure 7D–F), and tumor regression was observed in 50% of the mice (3 of 6) co‐treated with DNase I (Figure 7G). On Day 21 after the first injection, NK cell‐treatment resulted in a 71.7% reduction in the mean weight of SKAP1‐overexpressing tumors in DNase I‐treated mice, but only a 32.9% reduction in vehicle‐treated mice (Figure 7F). Similar results were observed in the SW620 colon tumor model where SKAP1 was knocked down instead of DNase I treatment (Figure 7H–J). In addition, NK cell therapy offered a greater survival benefit in mice with SKAP1‐knockdown SW620 tumors than in control tumor‐bearing mice. In the NK cell‐treated groups, SKAP1 knockdown significantly prolonged the survival of tumor‐bearing mice (Figure 7K). These results suggest that inhibition of SKAP1‐induced NET formation improves the efficacy of immunotherapy for colon cancer.

## 3 Discussion

In this study, we investigated the role of SKAP1, an immune cell adaptor protein, in colon cancer cells. Bulk RNA‐seq data analysis showed higher SKAP1 expression in colon cancer tissues compared to adjacent noncancerous tissues.^[^ ^8^ ^]^ Since SKAP1 is predominantly expressed in T lymphocytes,^[^ ^3^ ^]^ the elevated expression of SKAP1 revealed by bulk transcriptome analysis is possibly due to the increased density of infiltrating lymphocytes in colorectal cancer stroma compared to normal colonic mucosa.^[^ ^36^ ^]^ Herein, through immunohistochemistry and expression analysis in cell lines, we not only confirmed the elevated SKAP1 expression in colon cancer at the protein level, but also demonstrated that colon cancer cells indeed expressed abnormally high levels of SKAP1, suggesting its potential functional importance in colon cancer cells.

Previous immunological studies have demonstrated a critical role of SKAP1 in regulating “inside‐out” signaling during antigen‐induced T cell activation. Specifically, SKAP1 facilitates stable conjugation of T cells with antigen‐presenting cells, which is necessary for optimal T cell activation, by promoting the clustering of integrin lymphocyte‐associated antigen‐1 to increase the binding avidity for its ligand.^[^ ^4^ ^]^ In addition to mediating T cell adhesion, SKAP1 is also involved in the cycling of proliferating T cells.^[^ ^4^ ^]^ Although its functions in T cells have been extensively studied, the functional role of SKAP1 in cancer cells has not been well explored. Recently, SKAP1 was shown to promote the in vitro growth of gastric cancer cells^[^ ^9^ ^]^ and bladder cancer cells.^[^ ^37^ ^]^ Furthermore, SKAP1 enhanced the migratory and invasive abilities of gastric cancer cells.^[^ ^9^ ^]^ Regarding colorectal cancer, only one study provided in vitro data showing that SKAP1 knockdown in HT‐29 and HCT116 colon cancer cells inhibited cell proliferation, migration, and invasion.^[^ ^8^ ^]^ However, only a slight effect was observed in our in vitro experiments, possibly because of differences in SKAP1 expression modulation. We overexpressed rather than knocked down SKAP1 in HT‐29 and HCT116 cells because these two colon cancer cell lines expressed relatively low levels of SKAP1. Instead, we found a potent in vivo tumor‐promoting effect of SKAP1 in mice and consequently revealed a novel SKAP1‐induced cellular interaction between cancer cells and neutrophils, which largely accounted for its pro‐tumor activity. Our findings suggest a complex function of SKAP1 in colon cancer beyond the direct regulation of malignant behavior in cancer cells.

This study revealed that enhanced NET formation is an important neutrophil‐related mechanism contributing to the tumor‐promoting effect of SKAP1. NETs are crucial in mediating neutrophils’ pro‐tumor activity,^[^ ^23^ ^]^ with their principal component, DNA, direct promoting cancer cell proliferation.^[^ ^38^ ^]^ Our DNase‐based experiments confirmed the involvement of NET‐derived DNA in SKAP1‐induced colon cancer growth. Coiled‐coil domain containing protein 25 (CCDC25) serves as a NET‐DNA sensor on the surface of breast cancer cells.^[^ ^38^ ^]^ Given the high CCDC25 expression in colon cancers,^[^ ^38^ ^]^ it is plausible that SKAP1‐triggered NETs interact with CCDC25 to promote colon cancer cell proliferation. Recently, emerging evidence has suggested the immunosuppressive activity of NETs in the context of cancer and has shown improved efficacy of immune checkpoint blockade therapies after NET inhibition.^[^ ^39^ ^]^ NETs can shield tumor cells from immune‐mediated cytotoxicity by forming a physical barrier that obstructs contact between tumor cells and immune effector cells.^[^ ^28^ ^]^ Additionally, the NET‐mediated physical barrier contributes to T cell exclusion from tumors,^[^ ^40^ ^]^ aligning with the clinical observation that NET density was inversely correlated with CD8^+^ T cell densities in the tumor microenvironment.^[^ ^41^ ^]^ Beyond acting as a physical barrier, NETs may also induce the exhaustion and dysfunction of T cells,^[^ ^42^, ^43^ ^]^ impairing their antitumor functions. Here, we highlight the inhibitory effect of NETs on the immunocytotoxicity of NK cells, another crucial effector cell type involved in antitumor immune response. Notably, NET inhibition by SKAP1 downregulation in colon cancer cells markedly enhanced the therapeutic efficacy of adoptive NK cell transfer. Our results, together with those of a previous study showing that SKAP1 deficiency in T lymphocytes enhanced their antitumor immunity by suppressing PD‐1 expression,^[^ ^6^ ^]^ indicate that SKAP1 inhibition is a promising adjuvant approach for immune therapy in colon cancer, which warrants further investigation.

PMN‐MDSCs are considered as pathologically activated neutrophils in tumor‐bearing hosts, sharing origin and many morphological and phenotypic characteristics with classic neutrophils,^[^ ^20^ ^]^ such as cell‐surface expression of Ly6G. Consequently, distinguishing between neutrophils and PMN‐MDSCs remains challenging. Using CD14 as a marker for PMN‐MDSCs in tumor‐bearing mice,^[^ ^21^ ^]^ we showed that PMN‐MDSCs also accumulated in SKAP1‐overexpressing colon tumors. This may be due to increased infiltration of classical neutrophils, which can transform to CD14^+^ PMN‐MDSCs locally in the tumor microenvironment.^[^ ^21^ ^]^ PMN‐MDSCs can also create NETs^[^ ^44^ ^]^ and be eliminated by anti‐Ly6G antibody injection, therefore, we cannot discount the potential involvement of PMN‐MDSCs in SKAP1‐induced tumor promotion and NET formation. PMN‐MDSCs exert their immunosuppressive effects via multiple mechanisms.^[^ ^45^ ^]^ It is possible that a more complex mechanism underlies SKAP1‐induced immunosuppressive microenvironment, involving factors beyond NET formation.

CXCL8, rather than other neutrophil‐related cytokines, has been identified as the key mediator of SKAP1‐induced interactions between colon cancer cells and neutrophils. CXCL8 was initially identified as a monocyte‐derived neutrophil chemotactic factor.^[^ ^46^ ^]^ Currently, CXCL8 is believed to be also secreted by cancer cells, including colorectal cancer cells,^[^ ^47^ ^]^ and is involved in neutrophil migration into tumors.^[^ ^48^ ^]^ Our results suggest that SKAP1 is an upstream positive regulator of CXCL8 in colon cancer cells and is dependent on NFATc1, a crucial member of the NFAT family, to regulate CXCL8 expression. NFAT signaling plays an important role in the immune response by regulating the expression of inflammatory genes, including CXCL8.^[^ ^49^ ^]^ We extended this regulatory mechanism to colon cancer cells and characterized a novel SKAP1/NFATc1/CXCL8 axis that facilitated tumor growth. Notably, SKAP1 regulated the transcriptional expression of NFATc1 in colon cancer cells. A similar phenomenon was also observed in T lymphocytes,^[^ ^6^ ^]^ raising the question of how SKAP1 induces NFATc1 expression. In addition to its role in “inside‐out” integrin signaling, SKAP1 positively regulates the extracellular signal‐regulated kinase (ERK) pathway in T cells.^[^ ^50^ ^]^ Concurrently, NFATc1 serves as a downstream molecule of ERK signaling during osteoclast formation.^[^ ^51^ ^]^ These findings hint at ERK signaling as a potential intermediary between SKAP1 and NFATc1.

Although our study emphasizes the crucial role of the CXCL8‐NET axis, we cannot rule out other mechanisms involved in SKAP1‐induced tumor promotion. CXCL8, besides being a neutrophil chemotactic factor, also acts on endothelial cells and exerts angiogenic activity in the tumor microenvironment,^[^ ^52^ ^]^ suggesting that SKAP1 may promote tumor progression through the CXCL8‐dependent regulation of angiogenesis. Our cytokine array showed that SKAP1 also upregulated other secreted components, such as IL‐4, albeit to a lesser extent than CXCL8 (Figure S4A, Supporting Information). IL‐4, a classic anti‐inflammatory cytokine, can polarize macrophages to the M2 phenotype, thereby contributing to an immunosuppressive microenvironment for tumor growth.^[^ ^53^ ^]^ Additionally, SKAP1 overexpression conferred resistance against NK cell cytotoxicity even in the absence of neutrophils, although this resistance was markedly less pronounced than that in the presence of neutrophils. These results suggest that SKAP1 may affect the communication between cancer cells and immune cells, which facilitates colon tumor progression, via mechanisms independent of the CXCL8/NET axis.

Our study has several limitations. Nude mice models of human colon cancers have been used to study the in vivo interplay between colon cancer cells and neutrophils. Although human CXCL8 can act on the murine homologs of human CXCL8 receptors, the limitation of the species difference between tumor cells and neutrophils must be considered. Future studies are necessary to confirm the SKAP1‐induced interaction between colon cancer cells and neutrophils in human clinical samples. Clinical validation of the SKAP1/NFATc1/CXCL8 axis in colon cancer is also required. In addition, the present study did not clarify the precise molecular mechanisms underlying SKAP1‐induced translational upregulation of NFATc1, which warrant further in‐depth investigation.

## 4 Conclusion

**Figure 8:**
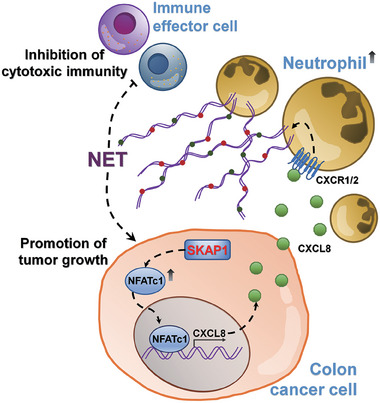
Schematic illustrating the SKAP1‐induced tumor‐promoting mechanisms in colon cancer. SKAP1 is significantly upregulated in colon cancer cells, leading to increased NFATc1‐dependent CXCL8 expression. This recruits neutrophils into the tumor microenvironment and promotes NET formation. SKAP1‐induced NETs potently enhance tumor growth and impair the cytotoxicity of immune effector cells against colon cancer cells.

The current study emphasizes the pro‐tumor role of SKAP1 in colon cancer cells. We provide evidence, for the first time, that SKAP1 expression in cancer cells potently promotes the in vivo growth of colon tumors and further revealed that the cancer cell/neutrophil NFATc1/CXCL8/NET axis is the key mechanism contributing to the tumor‐promoting effect of SKAP1 (**Figure** **8**). These findings indicate SKAP1 signaling as a promising target for colon cancer treatment. Given that anti‐CXCL8^[^ ^54^ ^]^ and anti‐NET therapies^[^ ^55^ ^]^ are currently being evaluated in clinical trials for the treatment of solid tumors, our study also proposes SKAP1 as a novel biomarker to identify patients with colon cancer who may benefit from these therapies.

## 5 Experimental Section

### Bioinformatics Analyses of Public Databases

The “Most Differential Survival Gene” function of GEPIA2 (http://gepia2.cancer‐pku.cn/)^[^ ^18^ ^]^ to generate a list of the top 100 genes that were mostly associated with the disease‐free survival of colon cancer patients based on TCGA database (group cutoff: top quartile versus bottom quartile) was used. It was also downloaded gene expression profiles and clinical data for patients with colon cancer from TCGA database and performed multivariate overall survival analysis using a Cox regression model. The mRNA expression levels of SKAP1 in colon cancer and noncancerous tissues were analyzed using data from TCGA and the GEO (https://www.ncbi.nlm.nih.gov/geo/) dataset GSE156614. To determine SKAP1 expression in cancer cell lines, the mRNA expression values of SKAP1 were obtained from the “Cell Line” section of The Human Protein Atlas portal (https://www.proteinatlas.org/). In addition, the TIMER algorithm^[^ ^56^ ^]^ was applied to estimate the enrichment scores of immune cells in colon cancers with high (above median) and low (below median) SKAP1 expression by analyzing TCGA data using the “immunedeconv” R package,^[^ ^57^ ^]^ which was implemented in R version 4.0.3.

### Tissue Microarray

To determine SKAP1 protein expression, a commercially available tissue microarray containing 94 cancerous and 86 noncancerous colon tissues (#HColA180Su21, Shanghai Outdo Biotech CO. Ltd., Shanghai, China) was subjected to immunohistochemical analysis using an anti‐SKAP1 antibody (Table S2, Supporting Information). Tissue samples were collected from Taizhou Hospital (Taizhou, Zhejiang Province, China) between 2012 and 2014 (demographic and clinical characteristics are listed in Table S3, Supporting Information), following approval from the Research Ethics Committees of Taizhou Hospital and Shanghai Outdo Biotech Company (YB M‐05‐02). Written informed consent was obtained from all patients. Quantitative analysis of SKAP1 staining in normal or neoplastic colon epithelial cells was performed according to a method described previously.^[^ ^58^ ^]^

### Cell Culture

Human colon cancer cell lines HT‐29, SW620, HCT116, and LoVo were obtained from the American Type Culture Collection. The normal colonic epithelial cell line NCM460 and human myeloid leukemia cell line HL‐60 were obtained from the Cell Bank of the Chinese Academy of Sciences (Shanghai, China). The IL‐2‐independent human NK cell line, NK‐92MI, was purchased from MingZhou Bio Co. (Ningbo, Zhejiang Province, China). HCT116 and HT‐29 cells were maintained in McCoy's 5 A medium, LoVo cells in F‐12K medium, SW620 cells in Leibovitz's L‐15 medium, and NCM460 cells in DMEM. These cell lines were cultured in media supplemented with 10% FBS. HL‐60 cells were grown in IMEM supplemented with 20% FBS and treated with 1.5% dimethyl sulfoxide and 25 ng/ml granulocyte colony‐stimulating factor (#300‐03, PeproTech) for four days to induce differentiation toward a neutrophilic phenotype. NK‐92MI cells were cultured in MEMα containing 12.5% FBS, 12.5% horse serum, 0.2 mM inositol, 0.02 mM folic acid, and 0.1 mM β‐mercaptoethanol. Cells were cultured in a 5% CO_2_ atmosphere at 37 °C for fewer than 20 passages and regularly tested for mycoplasma contamination using the MycoStrip Mycoplasma Detection Kit (#rep‐mysnc‐50, InvivoGen).

### Western Blot

Cell lysates were prepared and subjected to western blot analysis as previously described.^[^ ^59^ ^]^ The primary antibodies used were listed in Table S2 (Supporting Information). Western blot bands were quantified using Fiji (ImageJ2) open‐access software.

### Quantitative PCR

RNA isolation, reverse transcription, and quantitative PCR were performed as described previously.^[^ ^60^ ^]^ The relative expression levels of target genes were normalized to those of *GAPDH*. Primer sequences were listed in Table S4 (Supporting Information).

### Gene Overexpression and Knockdown

To stably overexpress SKAP1, HCT116 and HT‐29 cells were infected with a recombinant lentivirus carrying the human SKAP1 gene, purchased from GeneChem Co. (Shanghai, China), according to the manufacturer's protocol. Empty virus‐infected cells were used as controls. Stable SKAP1‐knockout SW620 cells were generated according to a previously described procedure^[^ ^58^ ^]^ using the CRISPR/Cas9 plasmid pSpCas9(BB)−2AGFP (PX458), a gift from Feng Zhang (#48 138, Addgene).^[^ ^61^ ^]^ The single guide RNA (sgRNA) sequences were shown in Table S4 (Supporting Information). To knock down different transcription factors, SKAP1‐overexpressing colon cells or control cells were transfected with plasmids expressing gene‐specific short hairpin RNAs (shRNA) or control shRNA using Lipofectamine 3000 (#L3000008, Invitrogen). All shRNA‐expressing plasmids were retrieved from an arrayed human TRC shRNA library (#RHS4012, Open Biosystems), and the shRNA sequences were shown in Table S4 (Supporting Information).

### Cell Proliferation Assay

Colon cells were seeded into 96‐well culture plates at a density of 3 × 10^3^ cells well^−1^ and incubated for the indicated times at 37 °C. Cell viability was monitored using a CCK‐8 kit (#CK04‐11, Dojindo) according to the manufacturer's instructions. To assess colon cancer cell proliferation in the presence of neutrophils, neutrophil‐differentiated HL‐60 cells (7.5 × 10^3^ cells well^−1^) were added after allowing the colon cancer cells to establish firm adhesion on culture plates (10‐12 h post‐seeding). In some experiments, the co‐cultured cells were treated with 0.25 mg ml^−1^ Dnase I (#11 284 932 001, Roche) to degrade NETs. Following incubation for 72 h, the HL‐60 cells were removed by gently washing twice with FBS‐free medium, and the viability of the adherent colon cancer cells was then assessed.

### Transwell‐Based Cell Migration/Invasion Assay

Transwell migration and invasion assays were performed as previously described.^58^ ^]^ For the invasion assay, the upper sides of Transwell inserts were coated with 100 µl ice‐cold diluted Matrigel (≈200 µg ml^−1^; #356 234, Corning).

### Tumor Xenograft Models

To construct a subcutaneous colon cancer model, SKAP1‐overexpressing or control HCT116 or HT‐29 cells and SKAP1‐knockdown or control SW620 cells (5 × 10^6^ cells mouse^−1^) were resuspended in serum‐free medium and injected into the right flank of 5‐week‐old male BALB/c nude mice (Shanghai Model Organisms Center, Shanghai, China). Body weight and tumor volume (calculated as 1/2 × length × width^2^) were measured twice per week. Mice were sacrificed when the tumor volume exceeded 800 mm^3^ or body weight loss exceeded 20%. Survival was defined as the time to sacrifice, and survival curves were obtained from the tumor growth of individual mice in different groups. In certain experiments, mice were sacrificed 31 or 35 days after tumor inoculation, and the tumors were excised and weighed. For anti‐NET treatment, tumor‐bearing mice were intravenously injected with DNase I (50 µg mouse^−1^) every 3 days, starting 2 days after tumor implantation. For CXCL8 blockade, mice were intraperitoneally injected with reparixin (50 µg mouse^−1^; #HY‐15251, MedChem Express) every two days. Mice in the vehicle group were administered the same volume of saline.

For NK cell therapy, colon cancer xenograft models were established in NOD‐*Prkdc^scid^ Il2rγ^null^* (NPG) mice (Shanghai Model Organisms Center) using indicated colon tumor cells. When the mean tumor volume reached ≈75 mm^3^, mice bearing SKAP1‐overexpressing HCT116 tumors or SKAP1‐knockdown or control SW620 tumors were randomized into NK cell‐treated groups (peritumoral injections of 5 × 10^6^ NK‐92MI cells in 100 µl PBS twice per week for 3 or 4 weeks) and PBS‐treated group. In some experiments, mice were intravenously injected with DNase I (50 µg/mouse) at the same time. Tumor growth and survival of the tumor‐bearing mice were monitored as described above.

All mice were housed under specific pathogen‐free conditions. Animal experiments were performed according to protocols approved by the Institutional Animal Care and Use Committee of Renji Hospital (RJ2022‐1017).

### In Vivo Depletion of Ly6G^+^ Cells

For systemic depletion of Ly6G^+^ cells, mice were intraperitoneally injected with anti‐Ly6G antibody (200 µg mouse^−1^; #BE0075‐1, Bioxcell) or its corresponding isotype control two days before tumor cell implantation, followed by repeated injections every 3 days until the mice were sacrificed.

### Flow Cytometry

Single‐cell suspensions from mouse spleen or tumor tissues were prepared as previously described.^[^ ^62^ ^]^ Cells were first incubated with anti‐CD16/CD32 antibodies (#14‐0161‐82, eBioscience) to block Fc‐mediated binding and then stained with primary antibodies (Table S2, Supporting Information) on ice for 45 min. After staining, the cells were detected using a FACSCalibur flow cytometer (BD Biosciences) and analyzed using FlowJo software (Tree Star). In some experiments, CD45^+^ CD11b^+^ Ly6G^+^ cells were enriched by fluorescence‐activated cell sorting using a FACSAria III cell sorter (BD Biosciences) and subjected to quantitative PCR analysis of arginase 1.

### Detection of MPO‐DNA

MPO‐DNA complexes in mouse plasma, tumor homogenate supernatants, and cell cultures were detected using a capture ELISA as previously described.^[^ ^24^, ^63^ ^]^ An anti‐MPO antibody (#AF3667, R&D Systems) was used as the capture antibody, and a peroxidase‐labeled anti‐DNA primary antibody (component No.2 of the Cell Death Detection ELISA Kit; #11 774 425 001, Roche) was used for detection. Mouse plasma was obtained by centrifuging fresh blood samples anticoagulated with EDTA. Freshly collected tumors were homogenized in PBS containing 0.1% Tween 20, and after centrifugation, the protein concentrations in the supernatant were determined using BCA Protein Assay Kits (#23 227, Thermo Fisher Scientific). Samples were then adjusted to achieve consistent protein concentrations. Equal volumes of plasma and tumor homogenate samples were subjected to MPO‐DNA ELISA.

### Immunofluorescence Detection of NETs in Tumor‐Infiltrating Neutrophils

Tumor‐infiltrating neutrophils were isolated using Precoll density gradient centrifugation and immunomagnetic separation, following a reported protocol^[^ ^64^ ^]^ with some modification. Briefly, single‐cell suspensions from tumors were prepared as previously described.^[^ ^62^ ^]^ Cells were resuspended in 4 ml of 35% Percoll (#17 089 109, Cytiva) and carefully layered onto an equal volume of 80% Percoll. After centrifugation at 1000 × g for 25 min without a break, the 35%/80% interface was collected, washed, and resuspended in PBS containing 0.5% BSA and 2 mM EDTA. Neutrophils were then isolated using the anti‐Ly6G microbeads (#130‐120‐337, Miltenyi Biotec) according to the manufacturer's introductions. The purity of the neutrophil‐enriched faction was confirmed by flow cytometry (CD11b^+^ Ly6G^+^ cells, > 85%). The collected cells were immobilized on positively‐charged slides, fixed with 4% paraformaldehyde, and permeabilized with 0.1% TritonX‐100. After blocked with 5% donkey serum (#SL050, Solarbio), cells were stained with anti‐Cit‐H3 and anti‐MPO antibodies overnight at 4 °C, followed by fluorescence‐labeled second antibodies for 2 h at room temperature. Finally, slides were counterstained with DAPI (#M5106, AbMole) and observed under a Leica TCS SP8 fluorescence microscope (Leica). Antibody information was provided in Table S2 (Supporting Information).

### In Vitro NETosis Assay and Cell‐Free DNA Detection

For NETosis induction, neutrophil‐differentiated HL‐60 cells were cultured with CM from the indicated colon cancer cells in the presence or absence of 100 nM reparixin, 0.2 µg ml^−1^ CXCL8‐neutralizing antibody (#MAB208, R&D), or 10 µM VIVIT (#M1261, AbMole). After 4 h of incubation, cell‐free double‐stranded DNA (dsDNA) in the culture supernatants was quantified using PicoGreen dsDNA Reagent (#P7581, Invitrogen) using a Synergy 2 microplate reader (BioTek). To detect NET‐forming cells, SYTOX Green^+^ HL‐60 cells were analyzed using flow cytometry.

### Cytokine Array

Eighty percent confluent HCT116‐EV or HCT116‐SKAP1ov cell cultures were washed three times with PBS, and then fresh medium was added. After 36 h of incubation, the CM was collected and subjected to semi‐quantitative detection of 80 cytokines using The Human Cytokine Antibody Array C5 (#AAH‐CYT‐5, RayBiotech), according to the manufacturer's instructions. Intensity units were detected by the chemiluminescence method and analyzed using Fiji software. The relative quantity of each protein was normalized to that of the positive controls included in the array.

### ELISA

Freshly collected tumor tissues were homogenized and lysed in 1× Lysis Buffer (#AA‐LYS‐10 ml, RayBiotech), and total proteins were quantified using BCA Protein Assay Kits. CXCL8 concentrations in tumor lysates and cell culture supernatants were determined using an IL‐8 ELISA kit (#ELH‐IL8‐1, RayBiotech).

### Cytotoxicity Assays

To assess NK cell‐mediated cytotoxicity in the presence of neutrophils, HCT116 cells were stained with the cell tracker DiR (#HY‐D1048, MedChem Express) and DiR‐labeled HCT116 cells were cultured in ultra‐low attachment 24‐well plate (0.5×10^6^ cells well^−1^; #174 930, Thermo Fisher Scientific) for 12 h to generate tumor spheroids. For cytotoxicity assays, neutrophil‐differentiated HL‐60 cells were cultured with CM from SKAP1‐overexpressing or control HCT116 cells for 4 h, and then added to 24‐well plates (1×10^6^ cells well^−1^), which contained preformed tumor spheroids, together with or without 0.25 mg/ml DNase I (Roche). Two hours later, co‐cultures were collected and incubated with 1×10^6^ NK‐92MI cells for additional 6 h. Afterward, the tumor spheroids were washed and trypsinized to single cells for propidium iodide (PI, #P1304MP, Thermo Fisher Scientific) staining. Dead HCT116 cells were determined by flow cytometric analysis of PI and DiR dual‐positive cells.

To assess the direct influence of SKAP1 overexpression on NK cell‐mediated cytotoxicity, 1 × 10^4^ SKAP1‐overexpressing or control HCT116 cells were co‐cultured with NK92‐MI cells in 96‐well microplates at effector‐to‐target ratios of 1:1 and 2:1, respectively. After incubation for 6 h, lactate dehydrogenase (LDH) levels in the supernatants were measured using a Cytotoxicity LDH Assay Kit (#CK12, Dojindo).

### Statistical Analysis

Data are presented as the mean ± SEMs or as box‐and‐whisker plots of medians with 10–90 percentiles. Statistical significance between two groups was analyzed using Student's *t* test or analysis of variance (ANOVA) unless stated otherwise. Survival curves were plotted using the Kaplan‒Meier method and compared using the log‐rank test. The significance threshold was set at *P* < 0.05 for all analyses.

## Conflict of Interest

The authors declare no conflict of interest.

## Author Contributions

J.G. and J.L. contributed equally to this work and shared the first authorship. S.W., Y.T., and Y.G. contributed to study conception and design. J. G. and J. Liu performed the experiments with assistance from J.Lu, X.Z., Q.L. and J.C. W.Z. and M.L. performed the bioinformatics analyses. J.G., J.Liu, and S.W. analyzed the data and wrote the manuscript. Y.T., Y.G., and Q.L. contributed to the constructive discussions and revised the manuscript. S.W. and Y.T. supervised the study. All authors have read and approved the final manuscript.

## Supporting information



Supporting Information

## Data Availability

The data that support the findings of this study are available from the corresponding author upon reasonable request.
