# Supplementary material for: SKAP1 Expression in Cancer Cells Enhances Colon Tumor Growth and Impairs Cytotoxic Immunity by Promoting Neutrophil Extracellular Trap Formation via the NFATc1/CXCL8 Axis
Source: Adv Sci (Weinh). 2024 Sep 13;11(41):2403430. doi: 10.1002/advs.202403430 (PMC11538704; doi:10.1002/advs.202403430)
Supplement: Supplementary file 1 — Supporting Information [file ADVS-11-2403430-s001.pdf]

ADVANCED SCIENCE

Open Access

Supporting Information

for *Adv. Sci.*, DOI 10.1002/advs.202403430

SKAP1 Expression in Cancer Cells Enhances Colon Tumor Growth and Impairs Cytotoxic Immunity by Promoting Neutrophil Extracellular Trap Formation via the NFATc1/CXCL8 Axis

Jian Gao, Jun Liu, Jilin Lu, Xiaofei Zhang, Wei Zhang, Qian Li, Jiayi Cai, Mengjun Li, Yu Gan, Yifan Tang and Shuangjie Wu**

Supporting Information

SKAP1 expression in cancer cells enhances colon tumor growth and impairs cytotoxic immunity by promoting neutrophil extracellular trap formation via the NFATc1/CXCL8 axis

*Jian Gao[1, #], Jun Liu[2, #], Jilin Lu[2], Xiaofei Zhang[2], Wei Zhang[1], Qian Li[1], Jiayi Cai[3], Mengjun Li[2], Yu Gan[1], Yifan Tang[2, *], Shuangjie Wu[2, *]*

J. Gao, W. Zhang, Q. Li, Y. Gan

[1] State Key Laboratory of Systems Medicine for Cancer, Shanghai Cancer Institute, Renji Hospital, Shanghai Jiao Tong University School of Medicine, Shanghai, China.

J. Liu, J. Lu, X. Zhang, M. Li, Y. Tang, S. Wu

[2] Department of General Surgery, Huashan Hospital (Hongqiao Campus), Fudan University, Shanghai, China

*Corresponding authors, E-mail: tangyifan@huashan.org.cn; wushuangjie@huashan.org.cn

J. Cai

[3] Clinical Research Unit, Renji Hospital, Shanghai Jiao Tong University School of Medicine, Shanghai, China

[#]**Equal contribution:** Jian Gao and Jun Liu contributed equally to this work.

Supplementary Figures

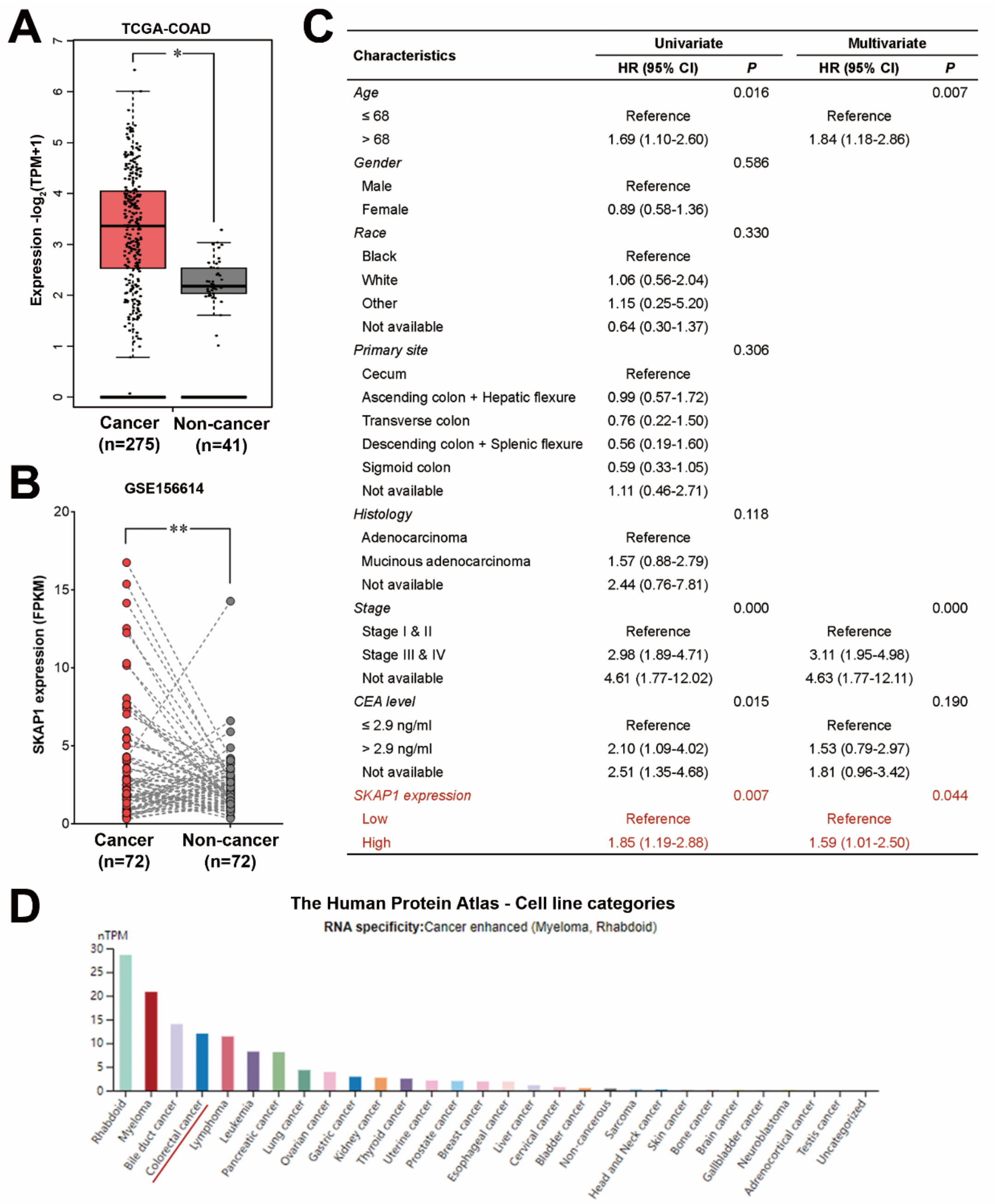

Characteristics	Univariate		Multivariate	
	HR (95% CI)	*P*	HR (95% CI)	*P*
Age		0.016		0.007
≤ 68	Reference		Reference	
> 68	1.69 (1.10-2.60)		1.84 (1.18-2.86)	
Gender		0.586		
Male	Reference			
Female	0.89 (0.58-1.36)			
Race		0.330		
Black	Reference			
White	1.06 (0.56-2.04)			
Other	1.15 (0.25-5.20)			
Not available	0.64 (0.30-1.37)			
Primary site		0.306		
Cecum	Reference			
Ascending colon + Hepatic flexure	0.99 (0.57-1.72)			
Transverse colon	0.76 (0.22-1.50)			
Descending colon + Splenic flexure	0.56 (0.19-1.60)			
Sigmoid colon	0.59 (0.33-1.05)			
Not available	1.11 (0.46-2.71)			
Histology		0.118		
Adenocarcinoma	Reference			
Mucinous adenocarcinoma	1.57 (0.88-2.79)			
Not available	2.44 (0.76-7.81)			
Stage		0.000		0.000
Stage I & II	Reference		Reference	
Stage III & IV	2.98 (1.89-4.71)		3.11 (1.95-4.98)	
Not available	4.61 (1.77-12.02)		4.63 (1.77-12.11)	
CEA level		0.015		0.190
≤ 2.9 ng/ml	Reference		Reference	
> 2.9 ng/ml	2.10 (1.09-4.02)		1.53 (0.79-2.97)	
Not available	2.51 (1.35-4.68)		1.81 (0.96-3.42)	
SKAP1 expression		0.007		0.044
Low	Reference		Reference	
High	1.85 (1.19-2.88)		1.59 (1.01-2.50)	

Supplementary Figure S1. SKAP1 is upregulated in colon cancer and independently predicts poor overall survival in colon cancer patients. **A,** Analysis of SKAP1 mRNA expression in colon cancer tissues and adjacent noncancerous tissues based on data from TCGA database. **B,** SKAP1 mRNA expression in colorectal cancer tissues and paired noncancerous tissues in the GEO dataset GSE156614. **C,** Cox regression analysis in colon cancer patients from the TCGA cohort. Note that multivariate analysis revealed high SKAP1 expression as an independent factor for poor overall survival in patients with colon cancer. **D,** *SKAP1* mRNA expression in various cell lines based on the data from the Human Protein Atlas website (https://www.proteinatlas.org/ENSG00000141293-SKAP1/cell+line). **, $P < 0.01$; *, $P < 0.05$.

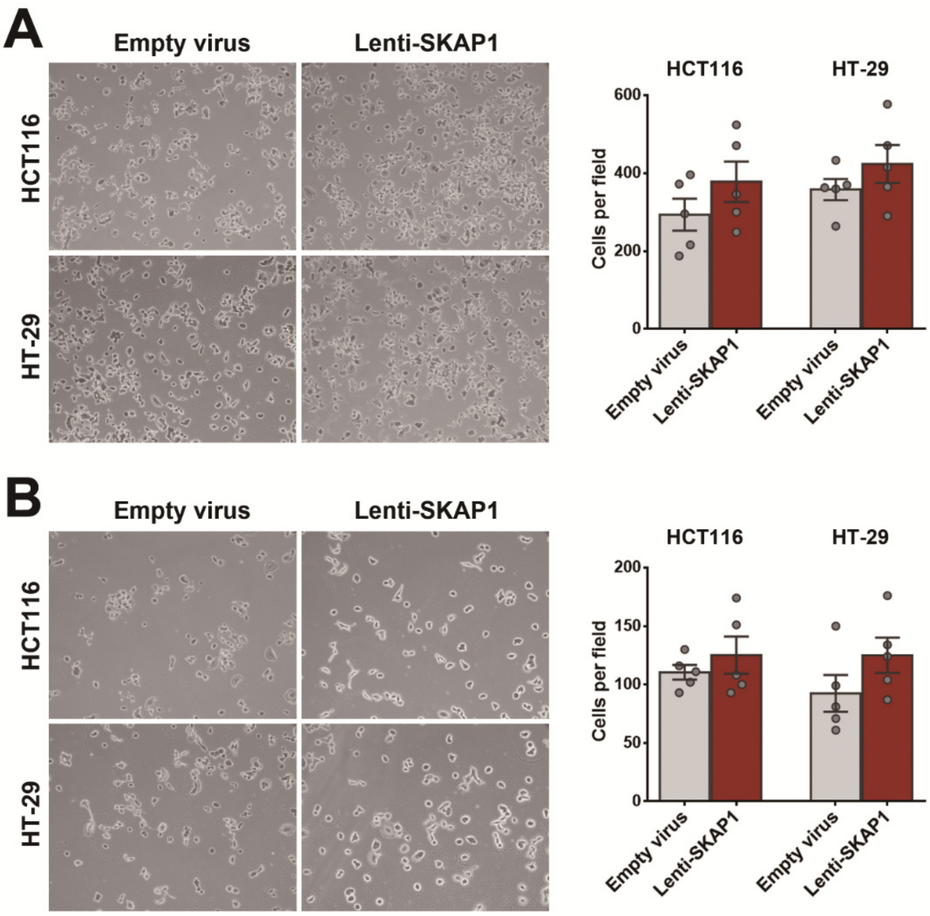

Supplementary Figure S2. SKAP1 overexpression does not influence the *in vitro* migration and invasion of colon cancer cells. **A,** The migratory abilities of HCT116 and HT-29 cells infected with a SKAP1-overexpressing lentivirus (Lenti-SKAP1) or empty virus were assessed using a Transwell-based migration assay. Representative images of crystal violet-stained Transwell membranes are shown on the left (original magnification, ×200). **B,** The invasion abilities of the same colon cells were evaluated using a Transwell-based invasion assay. Representative images of Matrigel-coated Transwell membrane stained with crystal violet are shown on the left (original magnification, ×200). Data are presented as the mean ± SEM.

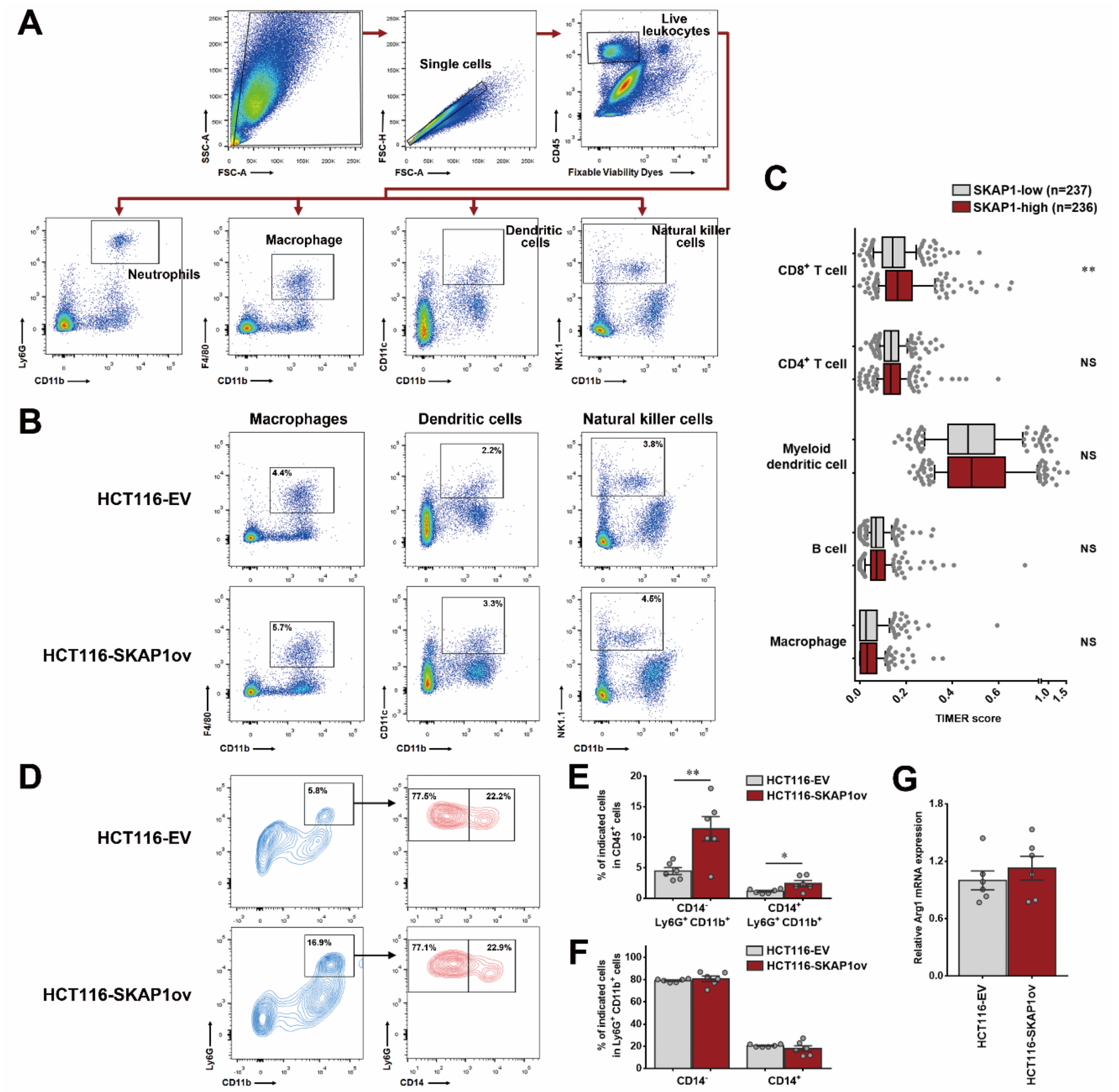

Supplementary Figure S3. SKAP1 overexpression promotes neutrophil infiltration into colon tumors. Tumor-infiltrating immune cells in SKAP1-overexpressing (HCT116-SKAP1ov) or control HCT116 tumors (HCT116-EV) were analyzed by flow cytometry. **A,** Gating strategy employed to analyze neutrophils ($CD45^+$ $CD11b^+$ $Ly6G^+$), macrophages ($CD45^+$ $CD11b^+$ $F4/80^+$), dendritic cells ($CD45^+$ $CD11b^+$ $CD11c^+$), and natural killer cells ($CD45^+$ $NK1.1^+$). **B,** Representative flow cytometry panels for analysis of tumor-infiltrating macrophages, dendritic cells, and natural killer cells in HCT116-SKAP1ov or HCT116-EV tumors. **C,** TIMER scores of different immune cells for SKAP1-high or SKAP1-low colon cancers from TCGA database. The *P* value was calculated using the Mann-Whitney test. **D-F,** Flow cytometry analysis of $CD14^-$ $CD11b^+$ $Ly6G^+$ and $CD14^+$ $CD11b^+$ $Ly6G^+$ cells in SKAP1-overexpressing (HCT116-SKAP1ov) or control (HCT116-EV) HCT116 tumors (n=6 per group). Representative flow cytometry panels are shown in **D**. **E,** Comparison of the proportions of $CD14^-$ $CD11b^+$ $Ly6G^+$ cells and $CD14^+$ $CD11b^+$ $Ly6G^+$ cells in tumor-infiltrating $CD45^+$ cells between groups. **F,** Comparison of the proportions of $CD14^-$ cells and $CD14^+$ cells in tumor-infiltrating $CD11b^+$ $Ly6G^+$ cells between groups. **G,** Quantitative PCR analysis of arginase 1 mRNA expression levels in tumor-infiltrating $CD11b^+$ $Ly6G^+$ cells from SKAP1-overexpressing or control HCT116 tumors. **E-G,** Data are presented as the mean ± SEM. **, $P < 0.01$; *, $P < 0.05$; NS, $P > 0.05$.

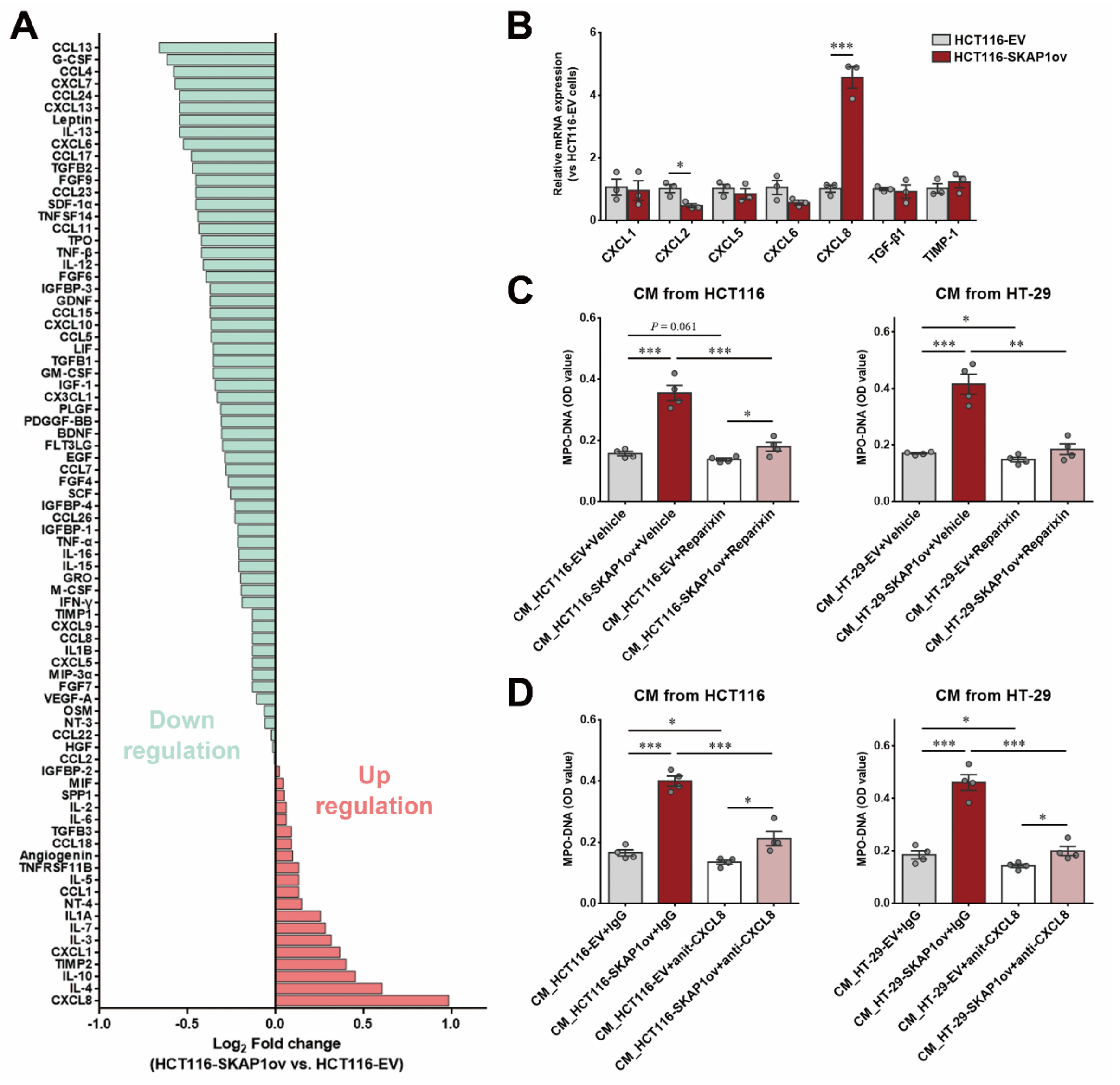

Supplementary Figure S4. SKAP1 expression in colon cancer cells promotes NET formation via CXCL8. **A,** Quantification of the cytokine array results shown in Figure 5A. Data are presented as fold change in the relative signal intensity for each protein. **B,** Quantitative PCR analysis of the mRNA expression levels of different cytokines related to neutrophil recruitment or NET formation in HCT116-SKAP1ov and HCT116-EV cells. **C-D,** Neutrophil-differentiated HL-60 cells were cultured with CM from SKAP1-overexpressing (HCT116-SKAP1ov or HT-29-SKAP1ov) or control cells (HCT116-EV or HT-29-EV) in the presence or absence of reparixin (**C**) or CXCL8-neutralizing antibody (**D**), and then MPO-DNA levels in culture supernatants were determined. Data are presented as the mean ± SEM. ***, $P < 0.001$; **, $P < 0.01$; *, $P < 0.05$.

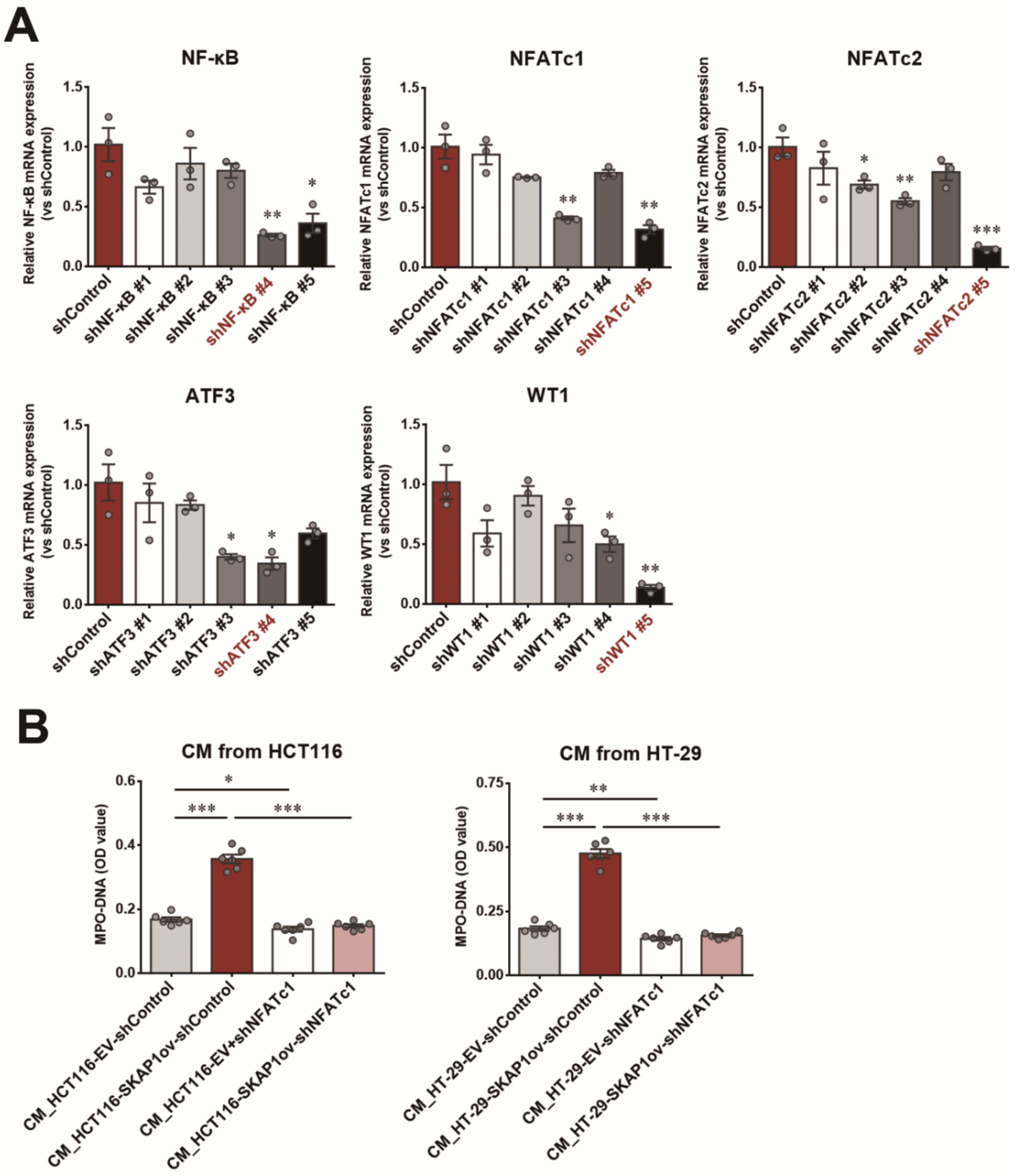

Supplementary Figure S5. SKAP1 in colon cancer cells promotes NET formation via NFATc1. **A,** Quantitative PCR analyses of the mRNA expression levels of the indicated transcription factors in HCT116 cells transfected with different shRNAs from a human TRC shRNA library. Five shRNAs were tested for each transcription factor, and those with the highest knockdown efficiency (shNF-κB #4, shNFATc1 #5, shNFATc2 #5, shATF3 #4, and shWT1 #5) were selected for subsequent experiments. **B,** Neutrophil-differentiated HL-60 cells were cultured with CM from SKAP1-overexpressing (HCT116-SKAP1ov or HT-29-SKAP1ov) or control cells (HCT116-EV or HT-29-EV) transfected with shNFATc1 or scrambled shRNA (shControl). MPO-DNA levels in culture supernatants were then measured. Data are presented as the mean ± SEM. ***, $P < 0.001$; **, $P < 0.01$; *, $P < 0.05$.

Supplementary Table S1. Top 100 genes with the lowest nominal P values in the analyses of the association between gene expression and disease-free survival in colon cancer.

Ensembl Gene ID*	Gene Name	Description	Hazard Ratio	P-value	-Log10(P-value)
ENSG00000266088	Novel gene	Novel transcript	4.6	1.27E-05	4.896196
ENSG00000246250	Novel gene	Novel transcript	4.8	6.34E-05	4.197911
ENSG00000250290	NCAPGP1	Non-SMC condensin I complex subunit G pseudogene 1	4.8	3.06E-04	3.514279
ENSG00000228157	Novel gene	Novel transcript	3.6	3.62E-04	3.441291
ENSG00000112561	TFEB	Transcription factor EB	4.1	4.12E-04	3.385103
ENSG00000279114	Novel gene	To be Experimentally Confirmed	5.6	4.32E-04	3.364516
ENSG00000120075	HOXB5	Homeobox B5	4	4.45E-04	3.351640
ENSG00000139182	CLSTN3	Calsyntenin 3	3.5	5.13E-04	3.289883
ENSG00000253846	PCDHGA10	Protocadherin gamma subfamily A, 10	3.3	5.13E-04	3.289883
ENSG00000172164	SNTB1	Syntrophin beta 1	3.3	5.84E-04	3.233587
ENSG00000168754	FAM178B	Family with sequence similarity 178 member B	3.9	7.18E-04	3.143876
ENSG00000115268	RPS15	Ribosomal protein S15	0.2	7.20E-04	3.142668
ENSG00000233680	HNRNPA1P27	Heterogeneous nuclear ribonucleoprotein A1 pseudogene 27	4.2	7.34E-04	3.134304
ENSG00000122547	EEPD1	Endonuclease/exonuclease/phosphatase family domain containing 1	3.5	7.39E-04	3.131356
ENSG00000279825	Novel gene	Novel transcript	3.7	7.52E-04	3.123782
ENSG00000141293	SKAP1	Src kinase associated phosphoprotein 1	3.4	7.73E-04	3.111821
ENSG00000271926	Novel gene	Novel transcript	3.3	7.84E-04	3.105684
ENSG00000263394	Novel gene	Novel transcript	3.1	7.86E-04	3.104577
ENSG00000055732	MCOLN3	Mucolipin TRP cation channel 3	3.2	8.69E-04	3.060980
ENSG00000239884	RN7SL608P	RNA, 7SL, cytoplasmic 608, pseudogene	3.5	9.22E-04	3.035269
ENSG00000274297	Novel gene	Novel transcript, sense intronic to UACA	4.1	9.52E-04	3.021363
ENSG00000154016	GRAP	GRB2 related adaptor protein	3	9.62E-04	3.016825
ENSG00000101197	BIRC7	Baculoviral IAP repeat containing 7	3.4	9.97E-04	3.001305
ENSG00000142609	CFAP74	Cilia and flagella associated protein 74	3.4	1.07E-03	2.970616
ENSG00000005471	ABCB4	ATP binding cassette subfamily B member 4	3.3	1.08E-03	2.966576
ENSG00000182742	HOXB4	Homeobox B4	3.1	1.10E-03	2.958607
ENSG00000111405	ENDOU	Endonuclease, poly(U) specific	3.1	1.18E-03	2.928118
ENSG00000161905	ALOX15	Arachidonate 15-lipoxygenase	3	1.18E-03	2.928118

ENSG00000236088	COX10-DT	COX10 divergent transcript	2.7	1.30E-03	2.886057
ENSG00000112511	PHF1	PHD finger protein 1	3.1	1.33E-03	2.876148
ENSG00000261596	Novel gene	Novel transcript, sense intronic to METTL9	3	1.37E-03	2.863279
ENSG00000204248	COL11A2	Collagen type XI alpha 2 chain	3.1	1.41E-03	2.850781
ENSG00000280157	Novel gene	To be Experimentally Confirmed	4	1.43E-03	2.844664
ENSG00000261488	TBILA	TGF-beta induced lncRNA	3.2	1.47E-03	2.832683
ENSG00000144130	NT5DC4	5'-nucleotidase domain containing 4	2.9	1.50E-03	2.823909
ENSG00000108511	HOXB6	Homeobox B6	3.3	1.56E-03	2.806875
ENSG00000163083	INHBB	Inhibin subunit beta B	2.8	1.56E-03	2.806875
ENSG00000164638	SLC29A4	Solute carrier family 29 member 4	2.8	1.56E-03	2.806875
ENSG00000234409	CCDC188	Coiled-coil domain containing 188	3.3	1.59E-03	2.798603
ENSG00000232926	RPL8P5	Ribosomal protein L8 (RPL8) pseudogene	3.4	1.64E-03	2.785156
ENSG00000241058	NSUN6	NOP2/Sun RNA methyltransferase 6	3.5	1.66E-03	2.779892
ENSG00000278743	Novel gene	Novel transcript	3.4	1.69E-03	2.772113
ENSG00000137218	FRS3	Fibroblast growth factor receptor substrate 3	3.2	1.69E-03	2.772113
ENSG00000185332	TMEM105	TMEM105 long non-coding RNA	3.2	1.69E-03	2.772113
ENSG00000134443	GRP	Gastrin releasing peptide	3.1	1.74E-03	2.759451
ENSG00000153291	SLC25A27	Solute carrier family 25 member 27	3.2	1.78E-03	2.749580
ENSG00000227097	RPS28P7	Ribosomal protein S28 pseudogene 7	0.32	2.03E-03	2.692504
ENSG00000227782	Novel gene	Novel transcript, antisense to NCOR1	3.6	2.04E-03	2.690370
ENSG00000180176	TH	Tyrosine hydroxylase	2.9	2.07E-03	2.684030
ENSG00000228335	Novel gene	Cytokine induced protein 29 kDa (CIP29) pseudogene	2.9	2.11E-03	2.675718
ENSG00000174951	FUT1	Fucosyltransferase 1 (H blood group)	3.2	2.12E-03	2.673664
ENSG00000173567	ADGRF3	Adhesion G protein-coupled receptor F3	3.4	2.17E-03	2.663540
ENSG00000279900	Novel gene	To be Experimentally Confirmed	3	2.23E-03	2.651695
ENSG00000125551	PLGLB2	Plasminogen like B2	2.9	2.28E-03	2.642065
ENSG00000212125	TAS2R15P	Taste 2 receptor member 15 pseudogene	3.4	2.38E-03	2.623423
ENSG00000262292	GRAPLDR	GRAPL long non-coding downstream RNA	2.9	2.43E-03	2.614394
ENSG00000166349	RAG1	Recombination activating 1	2.9	2.45E-03	2.610834
ENSG00000189223	PAX8-AS1	PAX8 antisense RNA 1	3.3	2.55E-03	2.593460
ENSG00000260027	HOXB7	Homeobox B7	3.2	2.72E-03	2.565431
ENSG00000144445	KANSL1L	KAT8 regulatory NSL complex subunit 1 like	3.1	2.73E-03	2.563837

ENSG00000276691	Novel gene	Novel transcript, antisense to HDAC7	3.1	2.77E-03	2.557520
ENSG00000100191	SLC5A4	Solute carrier family 5 member 4	3.5	2.78E-03	2.555955
ENSG00000182118	FAM89A	Family with sequence similarity 89 member A	2.7	2.82E-03	2.549751
ENSG00000262823	Novel gene	Novel transcript, antisense to SPNS3	0.28	2.96E-03	2.528708
ENSG00000273002	ARHGEF2-AS2	ARHGEF2 antisense RNA 2	3.1	2.97E-03	2.527244
ENSG00000228126	FALEC	Focally amplified long non-coding RNA in epithelial cancer	3	3.06E-03	2.514279
ENSG00000226252	Novel gene	Novel transcript	3.4	3.16E-03	2.500313
ENSG00000239552	HOXB-AS2	HOXB cluster antisense RNA 2	3.1	3.17E-03	2.498941
ENSG00000250615	Novel gene	Novel transcript	0.34	3.19E-03	2.496209
ENSG00000256690	STX5-DT	STX5 divergent transcript	3.2	3.26E-03	2.486782
ENSG00000176826	FKBP9P1	FKBP prolyl isomerase 9 pseudogene 1	2.8	3.26E-03	2.486782
ENSG00000182264	IZUMO1	Izumo sperm-oocyte fusion 1	3	3.29E-03	2.482804
ENSG00000126368	NR1D1	Nuclear receptor subfamily 1 group D member 1	3.1	3.32E-03	2.478862
ENSG00000184949	FAM227A	Family with sequence similarity 227 member A	2.7	3.48E-03	2.458421
ENSG00000108515	ENO3	Enolase 3	2.6	3.48E-03	2.458421
ENSG00000198753	PLXNB3	Plexin B3	2.6	3.53E-03	2.452225
ENSG00000280145	Novel gene	Novel transcript	3.3	3.55E-03	2.449772
ENSG00000273424	Novel gene	Novel transcript	3	3.59E-03	2.444906
ENSG00000230734	RPL10P3	Ribosomal protein L10 pseudogene 3	0.35	3.60E-03	2.443697
ENSG00000142549	IGLON5	IgLON family member 5	2.8	3.61E-03	2.442493
ENSG00000203684	IBA57-DT	IBA57 divergent transcript	3.2	3.66E-03	2.436519
ENSG00000109158	GABRA4	Gamma-aminobutyric acid type A receptor subunit alpha4	0.33	3.66E-03	2.436519
ENSG00000163395	IGFN1	Immunoglobulin like and fibronectin type III domain containing 1	3.2	3.71E-03	2.430626
ENSG00000187244	BCAM	Basal cell adhesion molecule	2.8	3.74E-03	2.427128
ENSG00000204380	PKP4-AS1	PKP4 antisense RNA 1	3.1	3.75E-03	2.425969
ENSG00000274561	Novel gene	Novel transcript	2.9	3.75E-03	2.425969
ENSG00000113389	NPR3	Natriuretic peptide receptor 3	3.1	3.83E-03	2.416801
ENSG00000183114	FAM43B	Family with sequence similarity 43 member B	3.8	3.88E-03	2.411168
ENSG00000121769	FABP3	Fatty acid binding protein 3	3	3.88E-03	2.411168
ENSG00000154102	C16orf74	Chromosome 16 open reading frame 74	3.1	4.03E-03	2.394695
ENSG00000118816	CCNI	Cyclin I	0.34	4.15E-03	2.381952
ENSG00000089169	RPH3A	Rabphilin 3A	3	4.16E-03	2.380907

ENSG00000261324	Novel gene	Novel transcript, overlapping HIST4H4	2.6	4.18E-03	2.378824
ENSG00000280164	Novel gene	Novel transcript	2.8	4.19E-03	2.377786
ENSG00000119684	MLH3	MutL homolog 3	3	4.20E-03	2.376751
ENSG00000196924	FLNA	Filamin A	3	4.21E-03	2.375718
ENSG00000148204	CRB2	Crumbs cell polarity complex component 2	2.6	4.24E-03	2.372634
ENSG00000225880	LINC00115	Long intergenic non-protein coding RNA 115	2.7	4.28E-03	2.368556
ENSG00000281530	DGCR12	DiGeorge syndrome critical region gene 12 (non-protein coding)	2.8	4.29E-03	2.367543
ENSG00000143248	RGS5	Regulator of G protein signaling 5	1.5	4.32E-03	2.364516

*Gene was excluded if its identifier is not present in the Ensembl GRCh38.p14.

Supplementary Table S2. Primary antibodies used in this study.

Antibody	Source	Cat. No.	Application	Dilution
Anti-SKAP1	Santa Cruz	sc-136068	Immunohistochemistry	1:100
			Western blot	1:200
Anti-GAPDH	Invitrogen	AM4300	Western blot	1:10,000
Anti-NFATc1	Santa Cruz	sc-7294	Western blot	1:200
Anti-Histone H3	Abcam	ab18521	Western blot	1:1000
Anti-CD45	BioLegend	103137	Flow cytometry	1:50
Anti-CD11b	Invitrogen	A15390	Flow cytometry	1:50
Anti-Ly6G	Invitrogen	46-9668-82	Flow cytometry	1:50
Anti-F4/80	BioLegend	123109	Flow cytometry	1:50
Anti-CD11c	BioLegend	117305	Flow cytometry	1:50
Anti-NK1.1	BioLegend	108713	Flow cytometry	1:50
Anti-CD14	eBioscience	12-0141-82	Flow cytometry	1:50
Anti-MPO	R&D system	AF3667	Immunofluorescence	1:200
Anti-Cit-H3	Abcam	ab281584	Immunofluorescence	1:200

Supplementary Table S3. Characteristics of the tissue samples included in tissue microarray.

Tissue No.	Gender	Age	Tissue type	Histology	Histological grade	Tumor site
D15A5131	Male	79	Cancerous & Paracancerous	Adenocarcinoma	II	Hepatic flexure of colon
D15A5133	Male	46	Cancerous & Paracancerous	Adenocarcinoma	II	Left-sided colon
D15A5139	Male	78	Cancerous & Paracancerous	Adenocarcinoma	II	Ileocecum
D15A5173	Male	69	Cancerous & Paracancerous	Adenocarcinoma	II-III	Sigmoid colon
D15A5144	Male	64	Cancerous & Paracancerous	Adenocarcinoma	II	Right-sided colon
D15A5146	Female	57	Cancerous & Paracancerous	Adenocarcinoma	II	Right-sided colon
D15A5147	Male	75	Cancerous & Paracancerous	Adenocarcinoma	II-III	Transverse colon
D15A5157	Male	65	Cancerous & Paracancerous	Adenocarcinoma	II-III	Left-sided colon
D15A5150	Female	70	Cancerous & Paracancerous	Adenocarcinoma	II	Right-sided colon
D15A5151	Female	77	Cancerous & Paracancerous	Mucinous adenocarcinoma	III	Ascending colon
D15A5152	Female	70	Cancerous & Paracancerous	Adenocarcinoma	II	Transverse colon
D15A5169	Female	57	Cancerous & Paracancerous	Signet ring cell carcinoma	III	Sigmoid colon
D15A6034	Male	63	Cancerous & Paracancerous	Adenocarcinoma	II-III	Descending colon
D15A5848	Male	48	Cancerous & Paracancerous	Adenocarcinoma	II	Left-sided colon
D15A5790	Male	78	Cancerous & Paracancerous	Adenocarcinoma	II	Sigmoid colon
D15A5793	Male	64	Cancerous & Paracancerous	Adenocarcinoma	II	Right-sided colon
D15A5797	Male	58	Cancerous & Paracancerous	Mucinous adenocarcinoma	III	Right-sided colon
D15A5794	Female	49	Cancerous & Paracancerous	Adenocarcinoma	II-III	Right-sided colon
D15A5795	Female	51	Cancerous & Paracancerous	Adenocarcinoma	II	Left-sided colon
D15A5796	Female	69	Cancerous & Paracancerous	Adenocarcinoma	II	Right-sided colon
D15A5792	Male	49	Cancerous & Paracancerous	Adenocarcinoma	II	Right-sided colon
D15A5875	Female	73	Cancerous & Paracancerous	Adenocarcinoma	II	Right-sided colon
D15A5800	Male	86	Cancerous & Paracancerous	Adenocarcinoma	II	Left-sided colon
D15A5851	Male	81	Cancerous & Paracancerous	Adenocarcinoma	II-III	Sigmoid colon
D15A5876	Female	80	Cancerous & Paracancerous	Adenocarcinoma	II	Right-sided colon

D15A5877	Male	60	Cancerous & Paracancerous	Mucinous adenocarcinoma	II-III	Descending colon
D15A5878	Female	67	Cancerous & Paracancerous	Adenocarcinoma	II	Right-sided colon
D15A5879	Female	73	Cancerous & Paracancerous	Adenocarcinoma	II-III	Right-sided colon
D15A5880	Male	39	Cancerous & Paracancerous	Adenocarcinoma	II-III	Left-sided colon
D15A5802	Female	77	Cancerous & Paracancerous	Adenocarcinoma	II	Sigmoid colon
D15A5803	Female	66	Cancerous & Paracancerous	Adenocarcinoma	II-III	Right-sided colon
D15A5804	Male	72	Cancerous & Paracancerous	Adenocarcinoma	II	Colon
D15A5805	Male	78	Cancerous & Paracancerous	Adenocarcinoma	II	Right-sided colon
D15A5806	Male	66	Cancerous & Paracancerous	Adenocarcinoma	II	Right-sided colon
D15A5808	Male	57	Cancerous & Paracancerous	Adenocarcinoma	II	Right-sided colon
D15A5882	Female	60	Cancerous & Paracancerous	Sarcomatoid carcinoma	III	Left-sided colon
D15A5809	Female	62	Cancerous & Paracancerous	Adenocarcinoma	II	Sigmoid colon
D15A5852	Male	44	Cancerous & Paracancerous	Adenocarcinoma	II	Descending colon
D15A5883	Female	79	Cancerous & Paracancerous	Adenocarcinoma	II	Right-sided colon
D15A5810	Female	72	Cancerous & Paracancerous	Adenocarcinoma	II-III	Descending colon
D15A5811	Male	57	Cancerous & Paracancerous	Adenocarcinoma	II	Right-sided colon
D15A5812	Male	51	Cancerous & Paracancerous	Adenocarcinoma	II-III	Sigmoid colon
D15A5813	Female	31	Cancerous & Paracancerous	Adenocarcinoma	II	Right-sided colon
D15A5884	Male	78	Cancerous & Paracancerous	Adenocarcinoma	II	Sigmoid colon
D15A5814	Female	74	Cancerous & Paracancerous	Adenocarcinoma	II	Sigmoid colon
D15A5816	Male	65	Cancerous & Paracancerous	Adenocarcinoma	II	Left-sided colon
D15A5815	Female	49	Cancerous & Paracancerous	Adenocarcinoma	II-III	Ascending colon
D15A5885	Male	51	Cancerous & Paracancerous	Adenocarcinoma	II	Sigmoid colon
D15A5817	Female	68	Cancerous & Paracancerous	Adenocarcinoma	II	Right-sided colon
D15A5818	Female	78	Cancerous & Paracancerous	Adenocarcinoma	II	Sigmoid colon
D15A5819	Female	57	Cancerous & Paracancerous	Adenocarcinoma	II	Descending colon
D15A5820	Female	60	Cancerous & Paracancerous	Mucinous adenocarcinoma	II-III	Colon

D15A6202	Female	68	Cancerous & Paracancerous	Adenocarcinoma	II	Sigmoid colon
D15A6191	Male	58	Cancerous & Paracancerous	Adenocarcinoma	II	Right-sided colon
D15A6192	Male	62	Cancerous & Paracancerous	Adenocarcinoma	II-III	Left-sided colon
D15A6194	Male	67	Cancerous & Paracancerous	Adenocarcinoma	II	Sigmoid colon
D15A6197	Female	78	Cancerous & Paracancerous	Adenocarcinoma	II	Colon
D15A6198	Male	46	Cancerous & Paracancerous	Adenocarcinoma	II	Sigmoid colon
D15A6199	Male	79	Cancerous & Paracancerous	Adenocarcinoma	II	Right-sided colon
D15A6201	Male	55	Cancerous & Paracancerous	Adenocarcinoma	II	Sigmoid colon
D15A6204	Female	56	Cancerous & Paracancerous	Adenocarcinoma	II	Sigmoid colon
D15A6205	Female	49	Cancerous & Paracancerous	Adenocarcinoma	II-III	Right-sided colon
D15A6206	Male	48	Cancerous & Paracancerous	Adenocarcinoma	II	Ileocecal valve
D15A6207	Female	79	Cancerous & Paracancerous	Adenocarcinoma	II	Sigmoid colon
D15A6208	Male	65	Cancerous & Paracancerous	Adenocarcinoma	II	Right-sided colon
D15A6209	Male	61	Cancerous & Paracancerous	Adenocarcinoma	II	Sigmoid colon
D15A6210	Female	40	Cancerous & Paracancerous	Adenocarcinoma	II-III	Sigmoid colon
D15A6211	Female	42	Cancerous & Paracancerous	Mucinous adenocarcinoma	II-III	Sigmoid colon
D15A6212	Female	57	Cancerous & Paracancerous	Mucinous adenocarcinoma	II-III	Colon
D15A6213	Female	71	Cancerous & Paracancerous	Adenocarcinoma	II	Right-sided colon
D15A6214	Male	80	Cancerous & Paracancerous	Adenocarcinoma	II	Sigmoid colon
D15A6217	Female	44	Cancerous & Paracancerous	Adenocarcinoma	II	Sigmoid colon
D15A6218	Male	54	Cancerous & Paracancerous	Adenocarcinoma	II	Right-sided colon
D15A6219	Female	81	Cancerous & Paracancerous	Adenocarcinoma	II-III	Ileocecal valve
D15A6220	Male	76	Cancerous & Paracancerous	Adenocarcinoma	II-III	Right-sided colon
D15A6221	Male	77	Cancerous & Paracancerous	Adenocarcinoma	III	Right-sided colon
D15A6222	Male	69	Cancerous & Paracancerous	Adenocarcinoma	II	Left-sided colon
D15A6224	Female	86	Cancerous & Paracancerous	Adenocarcinoma	II	Sigmoid colon
D15A6228	Female	75	Cancerous & Paracancerous	Adenocarcinoma	II	Colon

D15A6229	Female	76	Cancerous & Paracancerous	Adenocarcinoma	II-III	Colon
D15A6230	Female	65	Cancerous & Paracancerous	Adenocarcinoma	II-III	Right-sided colon
D15A6231	Female	55	Cancerous & Paracancerous	Adenocarcinoma	II-III	Right-sided colon
D15A6247	Female	83	Cancerous & Paracancerous	Adenocarcinoma	II	Sigmoid colon
D15A6248	Male	81	Cancerous & Paracancerous	Adenocarcinoma	II	Right-sided colon
D15A6249	Female	51	Cancerous & Paracancerous	Adenocarcinoma	II	Right-sided colon
D15A6252	Female	29	Cancerous & Paracancerous	Mucinous adenocarcinoma	III	Descending colon
D15A5140	Female	82	Cancerous	Adenocarcinoma	II-III	Right-sided colon
D15A5141	Male	71	Cancerous	Adenocarcinoma	II	Right-sided colon
D15A5874	Female	43	Cancerous	Adenocarcinoma	II-III	Sigmoid colon
D15A5801	Male		Cancerous	Adenocarcinoma	II	Left-sided colon
D15A6195	Male	68	Cancerous	Adenocarcinoma	II	Right-sided colon
D15A6200	Female	60	Cancerous	Mucinous adenocarcinoma	II-III	Ileocecum
D15A6253	Male	67	Cancerous	Adenocarcinoma	III	Right-sided colon
D15A6254	Female	61	Cancerous	Adenocarcinoma	II-III	Sigmoid colon

Supplementary Table S4. Primers, sgRNAs, and shRNAs used in this study.

Gene	Sequence (5'-3')	Application
Primer		
SKAP1	F: TCAAAGCCAGGTACTATTGGGA<br>R: AAAGGGTGCATCGGATGTGAG	Quantitative PCR
CXCL1	F: CTCTTCCGCTCCTCTCAC<br>R: GGGGACTTCACGTTCACACT	Quantitative PCR
CXCL2	F: AACATCCAAAGTGTGAAGGTGA<br>R: ATGCGGGGTTGAGACAAG	Quantitative PCR
CXCL5	F: AGCTGCGTTGCGTTTGTTTAC<br>R: TGGCGAACACTTGCAGATTAC	Quantitative PCR
CXCL6	F: AGAGCTGCGTTGCACTTGTT<br>R: GCAGTTTACCAATCGTTTTGGGG	Quantitative PCR
CXCL8	F: ACTGAGAGTGATTGAGAGTGGAC<br>R: AACCCTCTGCACCCAGTTTTC	Quantitative PCR
TGF-β1	F: CAATTCCTGGCGATACCTCAG<br>R: GCACAACTCCGGTGACATCAA	Quantitative PCR
TIMP-1	F: ACCACCTTATACCAGCGTTATGA<br>R: GGTGTAGACGAACCGGATGTC	Quantitative PCR
NFATc1	F: GCAGAGCACGGACAGCTATC<br>R: GGGCTTTCTCCACGAAAATGA	Quantitative PCR
ARG-1	F: CTCCAAGCCAAAGTCCTTAGAG<br>R: AGGAGCTGTCATTAGGGACATC	Quantitative PCR
sgRNA		
SKAP1	F: CACAGAGACCATATTCTACG<br>R: CGTAGAATATGGTCTCTGTG	CRISPR/Cas9-mediated SKAP1 knockout
shRNA		
NF-κB	#1: CGCCTGAATCATTCTCGATTT<br>#2: CCAGAGTTTACATCTGATGAT<br>#3: GCCTGAACAAATGTTTCATTT<br>#4: CCTTTCCTCTACTATCCTGAA<br>#5: CGAATGACAGAGGCGTGTATA	shRNA-mediated gene knockdown

NFATc1	#1: CGGCAACATTAGAAAGTGATT<br>#2: CCCGCCAACGTTCCAATTATA<br>#3: CGTCAGTTTCTACGTCTGCAA<br>#4: CATCGAGATAACCTCGTGCTT<br>#5: CGGAATCCTGAAACTCAGAAA	shRNA-mediated gene knockdown
NFATc2	#1: CCGAGTCCAAAGTTGTGTTTA<br>#2: CGCCAATAATGTCACCTCGAA<br>#3: GCACATCATGTACTGCGAGAA<br>#4: CCTCTTCGACTATGAGTATTT<br>#5: GTGAACTTCTACGTCATCAAT	shRNA-mediated gene knockdown
ATF3	#1: CCGCCTTTCATCTGGATTCTA<br>#2: GCATTTGATATACATGCTCAA<br>#3: CCTCTTTATCCAACAGATAAA<br>#4: CCTGAAGAAGATGAAAGGAAA<br>#5: GCTGAACTGAAGGCTCAGATT	shRNA-mediated gene knockdown
WT1	#1: GCAGCTAACAATGTCTGGTTA<br>#2: GCAGTGACAATTTATACCAAA<br>#3: CCAGGCTGCAATAAGAGATAT<br>#4: GCATCAGAGAAACATGACCAA<br>#5: GCATCTGAGACCAGTGAGAAA	shRNA-mediated gene knockdown
